# Stochastic discrete Hamiltonian variational integrators

**DOI:** 10.1007/s10543-018-0720-2

**Published:** 2018-08-16

**Authors:** Darryl D. Holm, Tomasz M. Tyranowski

**Affiliations:** 1 Mathematics Department, Imperial College London, London, SW7 2AZ UK; 2 Max-Planck-Institut für Plasmaphysik, Boltzmannstraße 2, 85748 Garching, Germany

**Keywords:** Stochastic Hamiltonian systems, Variational integrators, Geometric numerical integration methods, Geometric mechanics, Stochastic differential equations, 65C30

## Abstract

Variational integrators are derived for structure-preserving simulation of stochastic Hamiltonian systems with a certain type of multiplicative noise arising in geometric mechanics. The derivation is based on a stochastic discrete Hamiltonian which approximates a type-II stochastic generating function for the stochastic flow of the Hamiltonian system. The generating function is obtained by introducing an appropriate stochastic action functional and its corresponding variational principle. Our approach permits to recast in a unified framework a number of integrators previously studied in the literature, and presents a general methodology to derive new structure-preserving numerical schemes. The resulting integrators are symplectic; they preserve integrals of motion related to Lie group symmetries; and they include stochastic symplectic Runge–Kutta methods as a special case. Several new low-stage stochastic symplectic methods of mean-square order 1.0 derived using this approach are presented and tested numerically to demonstrate their superior long-time numerical stability and energy behavior compared to nonsymplectic methods.

## Introduction

Stochastic differential equations (SDEs) play an important role in modeling dynamical systems subject to internal or external random fluctuations. Standard references include [5, 27–29, 42, 50]. Within this class of problems, we are interested in stochastic Hamiltonian systems, which take the form (see [6, 30, 43])

1.1 $$\begin{aligned} dq&= \frac{\partial H}{\partial p}dt + \frac{\partial h}{\partial p}\circ dW(t), \nonumber \\ dp&= -\frac{\partial H}{\partial q}dt - \frac{\partial h}{\partial q}\circ dW(t), \end{aligned}$$

where $H=H(q,p)$ and $h=h(q,p)$ are the Hamiltonian functions, *W*(*t*) is the standard one-dimensional Wiener process, and $\circ$ denotes Stratonovich integration. The system (1.1) can be formally regarded as a classical Hamiltonian system with the randomized Hamiltonian given by $\widehat{H}(q,p) = H(q,p) + h(q,p)\circ {\dot{W}}$, where *H*(*q*, *p*) is the deterministic Hamiltonian and *h*(*q*, *p*) is *another* Hamiltonian, to be specified, which multiplies (in the Stratonovich sense, denoted as $\circ$) a one-dimensional Gaussian white noise, ${\dot{W}}$. Such systems can be used to model, e.g., mechanical systems with uncertainty, or error, assumed to arise from random forcing, limited precision of experimental measurements, or unresolved physical processes on which the Hamiltonian of the deterministic system might otherwise depend. Particular examples include modeling synchrotron oscillations of particles in particle storage rings (see [17, 56]) and stochastic dynamics of the interactions of singular solutions of the EPDiff basic fluids equation (see [23]). More examples are discussed in Sect. 4. See also [31, 37, 46, 54, 57, 58, 61].

As occurs for other SDEs, most Hamiltonian SDEs cannot be solved analytically and one must resort to numerical simulations to obtain approximate solutions. In principle, general purpose stochastic numerical schemes for SDEs can be applied to stochastic Hamiltonian systems. However, as for their deterministic counterparts, stochastic Hamiltonian systems possess several important geometric features. In particular, their phase space flows (almost surely) preserve the symplectic structure. When simulating these systems numerically, it is therefore advisable that the numerical scheme also preserves such geometric features. Geometric integration of deterministic Hamiltonian systems has been thoroughly studied (see [18, 41, 55] and the references therein) and symplectic integrators have been shown to demonstrate superior performance in long-time simulations of Hamiltonian systems, compared to non-symplectic methods; so it is natural to pursue a similar approach for stochastic Hamiltonian systems. This is a relatively recent pursuit. Stochastic symplectic integrators were first proposed in [43, 44]. Stochastic generalizations of symplectic partitioned Runge–Kutta methods were analyzed in [13, 35, 36]. A stochastic generating function approach to constructing stochastic symplectic methods, based on approximately solving a corresponding stochastic Hamilton–Jacobi equation satisfied by the generating function, was proposed in [65, 66], and this idea was further pursued in [2, 4, 16]. Stochastic symplectic integrators constructed via composition methods were proposed and analyzed in [45]. A first order weak symplectic numerical scheme and an extrapolation method were proposed and their global error was analyzed in [3]. More recently, an approach based on Padé approximations has been used to construct stochastic symplectic methods for linear stochastic Hamiltonian systems (see [60]). Higher-order strong and weak symplectic partitioned Runge–Kutta methods have been proposed in [67, 68]. High-order conformal symplectic and ergodic schemes for the stochastic Langevin equation have been introduced in [25]. Other structure-preserving methods for stochastic Hamiltonian systems have also been investigated, see, e.g., [1, 15, 26], and the references therein.

Long-time accuracy and near preservation of the Hamiltonian by symplectic integrators applied to deterministic Hamiltonian systems have been rigorously studied using the so-called backward error analysis (see, e.g., [18] and the references therein). To the best of our knowledge, such rigorous analysis has not been attempted in the stochastic context as yet. However, the numerical evidence presented in the papers cited above is promising and suggests that stochastic symplectic integrators indeed possess the property of very accurately capturing the evolution of the Hamiltonian *H* over exponentially long time intervals (note that the Hamiltonian *H* in general does not stay constant for stochastic Hamiltonian systems).

An important class of geometric integrators are *variational integrators*. This type of numerical schemes is based on discrete variational principles and provides a natural framework for the discretization of Lagrangian systems, including forced, dissipative, or constrained ones. These methods have the advantage that they are symplectic, and in the presence of a symmetry, satisfy a discrete version of Noether’s theorem. For an overview of variational integration for deterministic systems see [40]; see also [21, 32, 33, 47, 48, 53, 63, 64]. Variational integrators were introduced in the context of finite-dimensional mechanical systems, but were later generalized to Lagrangian field theories (see [39]) and applied in many computations, for example in elasticity, electrodynamics, or fluid dynamics; see [34, 49, 59, 62].

Stochastic variational integrators were first introduced in [8] and further studied in [7]. However, those integrators were restricted to the special case when the Hamiltonian function $h=h(q)$ was independent of *p*, and only low-order Runge–Kutta types of discretization were considered. In the present work we extend the idea of stochastic variational integration to general stochastic Hamiltonian systems by generalizing the variational principle introduced in [33] and applying a Galerkin type of discretization (see [32, 33, 40, 47, 48]), which leads to a more general class of stochastic symplectic integrators than those presented in [7, 8, 35, 36]. Our approach consists in approximating a generating function for the stochastic flow of the Hamiltonian system, but unlike in [65, 66], we do make this discrete approximation by exploiting its variational characterization, rather than solving the corresponding Hamilton–Jacobi equation.

*Main content* The main content of the remainder of this paper is, as follows.

- In Sect. 2 we introduce a stochastic variational principle and a stochastic generating function suitable for considering stochastic Hamiltonian systems, and we discuss their properties.
- In Sect. 3 we present a general framework for constructing stochastic Galerkin variational integrators, prove the symplecticity and conservation properties of such integrators, show they contain the stochastic symplectic Runge–Kutta methods of [35, 36] as a special case, and finally present several particularly interesting examples of new low-stage stochastic symplectic integrators of mean-square order 1.0 derived with our general methodology.
- In Sect. 4 we present the results of our numerical tests, which verify the theoretical convergence rates and the excellent long-time performance of our integrators compared to some popular non-symplectic methods.
- Section 5 contains the summary of our work.

## Variational principle for stochastic Hamiltonian systems

The stochastic variational integrators proposed in [7, 8] were formulated for dynamical systems which are described by a Lagrangian and which are subject to noise whose magnitude depends only on the position *q*. Therefore, these integrators are applicable to (1.1) only when the Hamiltonian function $h=h(q)$ is independent of *p* and the Hamiltonian *H* is non-degenerate (i.e., the associated Legendre transform is invertible). However, in the case of general $h=h(q,p)$ the paths *q*(*t*) of the system become almost surely nowhere differentiable, which poses a difficulty in interpreting the meaning of the corresponding Lagrangian. Therefore, we need a different sort of action functional and variational principle to construct stochastic symplectic integrators for (1.1). To this end, we will generalize the approach taken in [33]. To begin, in the next section, we will introduce an appropriate stochastic action functional and show that it can be used to define a type-II generating function for the stochastic flow of the system (1.1).

### Stochastic variational principle

Let the Hamiltonian functions $H: T^*Q \longrightarrow {\mathbb {R}}$ and $h: T^*Q \longrightarrow {\mathbb {R}}$ be defined on the cotangent bundle $T^*Q$ of the configuration manifold *Q*, and let (*q*, *p*) denote the canonical coordinates on $T^*Q$. For simplicity, in this work we assume that the configuration manifold has a vector space structure, $Q \cong {\mathbb {R}}^N$, so that $T^*Q = Q \times Q^* \cong {\mathbb {R}}^N \times {\mathbb {R}}^N$ and $TQ = Q \times Q \cong {\mathbb {R}}^N \times {\mathbb {R}}^N$. In this case, the natural pairing between one-forms and vectors can be identified with the scalar product on ${\mathbb {R}}^N$, that is, $\langle (q,p), (q,{\dot{q}}) \rangle = p\cdot {\dot{q}}$, where $(q,{\dot{q}})$ denotes the coordinates on *TQ* . Let $(\varOmega , \mathscr {F},\mathbb {P})$ be the probability space with the filtration $\{\mathscr {F}_t\}_{t \ge 0}$, and let *W*(*t*) denote a standard one-dimensional Wiener process on that probability space (such that *W*(*t*) is $\mathscr {F}_t$-measurable). We will assume that the Hamiltonian functions *H* and *h* are sufficiently smooth and satisfy all the necessary conditions for the existence and uniqueness of solutions to (1.1), and their extendability to a given time interval $[t_a, t_b]$ with $t_b > t_a \ge 0$. One possible set of such assumptions can be formulated by considering the Itô form of (1.1),

2.1 $$\begin{aligned} dz = A(z) dt + B(z)dW(t), \end{aligned}$$

with $$z=(q,p)$$ and

2.2 $$\begin{aligned} A(z) = \begin{pmatrix} \frac{\partial H}{\partial p}+\frac{1}{2}\frac{\partial ^2 h}{\partial p \partial q} \frac{\partial h}{\partial p}-\frac{1}{2}\frac{\partial ^2 h}{\partial p^2} \frac{\partial h}{\partial q} \\ -\frac{\partial H}{\partial q}-\frac{1}{2}\frac{\partial ^2 h}{\partial q^2} \frac{\partial h}{\partial p}+\frac{1}{2}\frac{\partial ^2 h}{\partial q \partial p} \frac{\partial h}{\partial q} \end{pmatrix}, \qquad B(z) = \begin{pmatrix} \frac{\partial h}{\partial p} \\ -\frac{\partial h}{\partial q} \end{pmatrix}, \end{aligned}$$

where $\partial ^2 h/\partial q^2$, $\partial ^2 h/\partial p^2$, and $\partial ^2 h/\partial q \partial p$ denote the Hessian matrices of *h*. For simplicity and clarity of the exposition, throughout this paper we assume that (see [5, 27–29])

1. *H* and *h* are $C^2$ functions of their arguments
2. *A* and *B* are globally Lipschitz

These assumptions are sufficient^1^ for our purposes, but could be relaxed if necessary. Define the space

2.3 $$\begin{aligned} C([t_a, t_b])= & {} \big \{ (q,p):\varOmega \times [t_a, t_b]\nonumber \\&\longrightarrow T^*Q \, \big | \, q, p \text { are almost surely continuous } \mathscr {F}_t\text {-adapted semimartingales} \big \}.\nonumber \\ \end{aligned}$$

Since we assume $T^*Q \cong {\mathbb {R}}^N \times {\mathbb {R}}^N$, the space $C([t_a, t_b])$ is a vector space (see [50]). Therefore, we can identify the tangent space $TC([t_a, t_b]) \cong C([t_a, t_b])\times C([t_a, t_b])$. We can now define the following stochastic action functional, $\mathscr {B}: \varOmega \times C([t_a, t_b]) \longrightarrow {\mathbb {R}}$,

2.4 $$\begin{aligned} \mathscr {B}\big [q(\cdot ),p(\cdot ) \big ] = p(t_b)q(t_b) - \int _{t_a}^{t_b} \Big [ p\circ dq - H\big (q(t),p(t)\big )\,dt - h\big (q(t),p(t)\big )\circ dW(t)\Big ], \end{aligned}$$

where $\circ$ denotes Stratonovich integration, and we have omitted writing the elementary events $\omega \in \varOmega$ as arguments of functions, following the standard convention in stochastic analysis.

#### Theorem 2.1

(Stochastic variational principle in phase space) Suppose that *H*(*q*, *p*) and *h*(*q*, *p*) satisfy conditions (H1)–(H2). If the curve $\big ( q(t), p(t) \big )$ in $T^*Q$ satisfies the stochastic Hamiltonian system (1.1) for $t\in [t_a,t_b]$, where $t_b \ge t_a >0$, then the pair $\big ( q(\cdot ), p(\cdot ) \big )$ is a critical point of the stochastic action functional (2.4), that is,

2.5 $$\begin{aligned} \delta \mathscr {B}\big [q(\cdot ), p(\cdot ) \big ] \equiv \frac{d}{d\epsilon } \bigg |_{\epsilon =0}\mathscr {B}\big [q(\cdot )+\epsilon \delta q(\cdot ), p(\cdot )+\epsilon \delta p(\cdot ) \big ]=0, \end{aligned}$$

almost surely for all variations $\big (\delta q(\cdot ), \delta p(\cdot ) \big ) \in C([t_a, t_b])$ such that almost surely $\delta q(t_a)=0$ and $\delta p(t_b)=0$.

#### Proof

Let the curve $\big ( q(t), p(t) \big )$ in $T^*Q$ satisfy (1.1) for $t\in [t_a,t_b]$. It then follows that the stochastic processes *q*(*t*) and *p*(*t*) are almost surely continuous, $\mathscr {F}_t$-adapted semimartingales, that is, $\big ( q(\cdot ), p(\cdot ) \big ) \in C([t_a, t_b])$ (see [5, 50]). We calculate the variation (2.5) as

2.6 $$\begin{aligned} \delta \mathscr {B}\big [q(\cdot ), p(\cdot ) \big ]&= p(t_b)\delta q(t_b) - \int _{t_a}^{t_b} p(t)\circ d \delta q(t) - \int _{t_a}^{t_b} \delta p(t)\circ d q(t) \nonumber \\&\quad + \int _{t_a}^{t_b} \bigg [ \frac{\partial H}{\partial q}\big (q(t),p(t)\big )\,\delta q(t) + \frac{\partial H}{\partial p}\big (q(t),p(t)\big )\,\delta p(t) \bigg ]\,dt \nonumber \\&\quad + \int _{t_a}^{t_b} \bigg [ \frac{\partial h}{\partial q}\big (q(t),p(t)\big )\,\delta q(t) + \frac{\partial h}{\partial p}\big (q(t),p(t)\big )\,\delta p(t) \bigg ]\circ dW(t), \end{aligned}$$

where we have used the end point condition, $\delta p(t_b)=0$. Since the Hamiltonians are $C^2$ and the processes *q*(*t*), *p*(*t*) are almost surely continuous, in the last two lines we have used a dominated convergence argument to interchange differentiation with respect to $\epsilon$ and integration with respect to *t* and *W*(*t*). Upon applying the integration by parts formula for semimartingales (see [50]), we find

2.7 $$\begin{aligned} \int _{t_a}^{t_b} p(t)\circ d \delta q(t) = p(t_b)\delta q(t_b) - p(t_a)\delta q(t_a) - \int _{t_a}^{t_b} \delta q(t)\circ d p(t). \end{aligned}$$

Substituting and rearranging terms produces,

2.8 $$\begin{aligned} \delta \mathscr {B}\big [q(\cdot ), p(\cdot ) \big ]&= \int _{t_a}^{t_b} \delta q(t) \bigg [\circ dp(t) + \frac{\partial H}{\partial q}\big (q(t),p(t)\big )\,dt + \frac{\partial h}{\partial q}\big (q(t),p(t)\big )\circ dW(t) \bigg ] \nonumber \\&\quad - \int _{t_a}^{t_b} \delta p(t) \bigg [\circ dq(t) - \frac{\partial H}{\partial p}\big (q(t),p(t)\big )\,dt - \frac{\partial h}{\partial p}\big (q(t),p(t)\big )\circ dW(t) \bigg ], \end{aligned}$$

where we have used $\delta q(t_a)=0$. Since $\big ( q(t), p(t) \big )$ satisfy (1.1), then by definition we have that almost surely for all $t \in [t_a,t_b]$,

2.9 $$\begin{aligned} q(t) = q(t_a) + \underbrace{\int _{t_a}^t \frac{\partial H}{\partial p}(q(s),p(s)) \,ds}_{M_1(t)} + \underbrace{\int _{t_a}^t \frac{\partial h}{\partial p}(q(s),p(s)) \circ dW(s)}_{M_2(t)}, \end{aligned}$$

that is, *q*(*t*) can be represented as the sum of two semi-martingales $M_1(t)$ and $M_2(t)$, where the sample paths of the process $M_1(t)$ are almost surely continuously differentiable. Let us calculate

2.10 $$\begin{aligned} \int _{t_a}^{t_b} \delta p(t)\circ dq(t)&= \int _{t_a}^{t_b} \delta p(t)\circ d\big (q(t_a)+M_1(t)+M_2(t)\big ) \nonumber \\&= \int _{t_a}^{t_b} \delta p(t)\circ dM_1(t) + \int _{t_a}^{t_b} \delta p(t)\circ dM_2(t) \nonumber \\&= \int _{t_a}^{t_b} \delta p(t)\frac{\partial H}{\partial p}(q(t),p(t)) \,dt + \int _{t_a}^{t_b} \delta p(t) \frac{\partial h}{\partial p}(q(t),p(t)) \circ dW(t), \end{aligned}$$

where in the last equality we have used the standard property of the Riemann–Stieltjes integral for the first term, as $M_1(t)$ is almost surely differentiable, and the associativity property of the Stratonovich integral for the second term (see [27, 50]). Substituting (2.10) in (2.8), we show that the second term is equal to zero. By a similar argument we also prove that the first term in (2.8) is zero. Therefore, $\delta \mathscr {B} = 0$, almost surely. $\square$

**Remark** It is natural to expect that the converse theorem, that is, if $\big ( q(\cdot ), p(\cdot ) \big )$ is a critical point of the stochastic action functional (2.4), then the curve $\big ( q(t), p(t) \big )$ satisfies (1.1), should also hold, although a larger class of variations $(\delta q, \delta p)$ may be necessary. A variant of such a theorem, although for a slightly different variational principle and in a different setting, was proved in Lázaro-Camí and Ortega [30]. Another variant for Lagrangian systems was proved by Bou-Rabee and Owhadi [8] in the special case when $h=h(q)$ is independent of *p*. In that case, one can assume that *q*(*t*) is continuously differentiable. In the general case, however, *q*(*t*) is not differentiable, and the ideas of [8] cannot be applied directly. We leave this as an open question. Here, we will use the action functional (2.4) and the variational principle (2.5) to construct numerical schemes, and we will directly verify that these numerical schemes converge to solutions of (1.1).

### Stochastic type-II generating function

When the Hamiltonian functions *H*(*q*, *p*) and *h*(*q*, *p*) satisfy standard measurability and regularity conditions [e.g., (H1)–(H2)], then the system (1.1) possesses a pathwise unique stochastic flow $F_{t,t_0}: \varOmega \times T^*Q \longrightarrow T^*Q$. It can be proved that for fixed $t,t_0$ this flow is mean-square differentiable with respect to the *q*, *p* arguments, and is also almost surely a diffeomorphism (see [5, 27–29]). Moreover, $F_{t,t_0}$ almost surely preserves the canonical symplectic form $\varOmega _{T^*Q} = \sum _{i=1}^N dq^i \wedge dp^i$ (see [6, 30, 44]), that is,

2.11 $$\begin{aligned} F^*_{t,t_0} \varOmega _{T^*Q} = \varOmega _{T^*Q}, \end{aligned}$$

where $F^*_{t,t_0}$ denotes the pull-back by the flow $F_{t,t_0}$. We will show below that the action functional (2.4) can be used to construct a type II generating function for $F_{t,t_0}$. Let $({\bar{q}}(t), {\bar{p}}(t))$ be a particular solution of (1.1) on $[t_a,t_b]$. Suppose that for almost all $\omega \in \varOmega$ there is an open neighborhood $\mathscr {U}(\omega )\subset Q$ of ${\bar{q}}(\omega ,t_a)$, an open neighborhood $\mathscr {V}(\omega )\subset Q^*$ of ${\bar{p}}(\omega ,t_b)$, and an open neighborhood $\mathscr {W}(\omega )\subset T^*Q$ of the curve $({\bar{q}}(\omega ,t), {\bar{p}}(\omega ,t))$ such that for all $q_a \in \mathscr {U}(\omega )$ and $p_b \in \mathscr {V}(\omega )$ there exists a pathwise unique solution $({\bar{q}}(\omega ,t; q_a, p_b), {\bar{p}}(\omega ,t; q_a, p_b))$ of (1.1) which satisfies ${\bar{q}}(\omega ,t_a; q_a, p_b)=q_a$, ${\bar{p}}(\omega ,t_b; q_a, p_b)=p_b$, and $({\bar{q}}(\omega ,t; q_a, p_b), {\bar{p}}(\omega ,t; q_a, p_b)) \in \mathscr {W}(\omega )$ for $t_a\le t \le t_b$. (As in the deterministic case, for $t_b$ sufficiently close to $t_a$ one can argue that such neighborhoods exist; see [38].) Define the function $S:\mathscr {Y} \longrightarrow {\mathbb {R}}$ as

2.12 $$\begin{aligned} S(q_a,p_b) = \mathscr {B}\big [{\bar{q}}(\cdot ;q_a,p_b), {\bar{p}}(\cdot ; q_a,p_b) \big ], \end{aligned}$$

where the domain $\mathscr {Y} \subset \varOmega \times T^*Q$ is given by $\mathscr {Y} = \bigcup \nolimits _{\omega \in \varOmega } \{\omega \} \times \mathscr {U}(\omega ) \times \mathscr {V}(\omega )$. Below we prove that *S* generates^2^ the stochastic flow $F_{t_b,t_a}$.

#### Theorem 2.2

The function $S(q_a,p_b)$ is a type-II stochastic generating function for the stochastic mapping $F_{t_b,t_a}$, that is, $F_{t_b,t_a}:(q_a,p_a)\longrightarrow (q_b,p_b)$ is implicitly given by the equations

2.13 $$\begin{aligned} q_b = D_2 S(q_a,p_b), \qquad p_a = D_1 S(q_a,p_b), \end{aligned}$$

where the derivatives are understood in the mean-square sense.

#### Proof

Under appropriate regularity assumptions on the Hamiltonians [e.g., (H1)–(H2)], the solutions ${\bar{q}}(t; q_a, p_b)$ and ${\bar{p}}(t; q_a, p_b)$ are mean-square differentiable with respect to the parameters $q_a$ and $p_b$, and the partial derivatives are semimartingales (see [5]). We calculate the derivative of *S* as

2.14 $$\begin{aligned} \frac{\partial S}{\partial q_a}(q_a,p_b)&= p_b \frac{\partial {\bar{q}}(t_b)}{\partial q_a} - \int _{t_a}^{t_b} \frac{\partial {\bar{p}}(t)}{\partial q_a} \circ d {\bar{q}}(t) - \int _{t_a}^{t_b} {\bar{p}}(t) \circ d \frac{\partial {\bar{q}}(t)}{\partial q_a} \nonumber \\&\quad + \int _{t_a}^{t_b} \bigg [ \frac{\partial {\bar{q}}(t)}{\partial q_a} \frac{\partial H}{\partial q} \big ({\bar{q}}(t), {\bar{p}}(t) \big ) + \frac{\partial {\bar{p}}(t)}{\partial q_a} \frac{\partial H}{\partial p} \big ({\bar{q}}(t), {\bar{p}}(t) \big ) \bigg ]\,dt \nonumber \\&\quad + \int _{t_a}^{t_b} \bigg [ \frac{\partial {\bar{q}}(t)}{\partial q_a} \frac{\partial h}{\partial q} \big ({\bar{q}}(t), {\bar{p}}(t) \big ) + \frac{\partial {\bar{p}}(t)}{\partial q_a} \frac{\partial h}{\partial p} \big ({\bar{q}}(t), {\bar{p}}(t) \big ) \bigg ]\circ dW(t), \end{aligned}$$

where for notational convenience we have omitted writing $q_a$ and $p_b$ explicitly as arguments of ${\bar{q}}(t)$ and ${\bar{p}}(t)$. Applying the integration by parts formula for semimartingales (see [50]), we find

2.15 $$\begin{aligned} \int _{t_a}^{t_b} {\bar{p}}(t) \circ d \frac{\partial {\bar{q}}(t)}{\partial q_a} = p_b\frac{\partial {\bar{q}}(t_b)}{\partial q_a} - {\bar{p}}(t_a) - \int _{t_a}^{t_b} \frac{\partial {\bar{q}}(t)}{\partial q_a} \circ d {\bar{p}}(t). \end{aligned}$$

Substituting and rearranging terms, we obtain the result,

2.16 $$\begin{aligned} \frac{\partial S}{\partial q_a}(q_a,p_b)&= {\bar{p}}(t_a) + \int _{t_a}^{t_b}\frac{\partial {\bar{q}}(t)}{\partial q_a} \bigg [\circ d {\bar{p}} + \frac{\partial H}{\partial q} \big ({\bar{q}}(t), {\bar{p}}(t) \big )\,dt + \frac{\partial h}{\partial q} \big ({\bar{q}}(t), {\bar{p}}(t) \big )\circ dW(t) \bigg ] \nonumber \\&\qquad + \int _{t_a}^{t_b}\frac{\partial {\bar{p}}(t)}{\partial q_a} \bigg [\circ d {\bar{q}} - \frac{\partial H}{\partial p} \big ({\bar{q}}(t), {\bar{p}}(t) \big )\,dt - \frac{\partial h}{\partial p} \big ({\bar{q}}(t), {\bar{p}}(t) \big )\circ dW(t) \bigg ] \nonumber \\&= {\bar{p}}(t_a), \end{aligned}$$

since $({\bar{q}}(t), {\bar{p}}(t))$ is a solution of (1.1). Similarly we show $\partial S / \partial p_b (q_a,p_b) = {\bar{q}}(t_b)$. By definition of the flow, then $F_{t_b,t_a}(q_a, {\bar{p}}(t_a)) = ({\bar{q}}(t_b), p_b)$. $\square$

We can consider $S(q_a,p_b)$ as a function of time if we let $t_b$ vary. Let us denote this function as $S_t(q_a,p)$. Below we show that $S_t(q_a,p)$ satisfies a certain stochastic partial differential equation, which is a stochastic generalization of the Hamilton–Jacobi equation considered in [33].

#### Proposition 2.1

(Type II stochastic Hamilton–Jacobi equation) Let the time-dependent type-II generating function be defined as

2.17 $$\begin{aligned} S_2(q_a,p,t)\equiv & {} S_t(q_a,p) \nonumber \\= & {} p {\bar{q}}(t) - \int _{t_a}^{t} \Big [ {\bar{p}}(\tau ) \circ d{\bar{q}}(\tau ) - H\big ({\bar{q}}(\tau ),{\bar{p}}(\tau )\big )\,d\tau - h\big ({\bar{q}}(\tau ),{\bar{p}}(\tau )\big )\circ dW(\tau )\Big ],\nonumber \\ \end{aligned}$$

where ${\bar{q}}(\tau ) \equiv {\bar{q}}(\tau ; q_a, p)$ and ${\bar{p}}(\tau ) \equiv {\bar{p}}(\tau ; q_a, p)$ as before. Then the function $S_2(q_a,p,t)$ satisfies the following stochastic partial differential equation

2.18 $$\begin{aligned} d S_2 = H\left( \frac{\partial S_2}{\partial p}, p \right) \,dt + h\left( \frac{\partial S_2}{\partial p}, p \right) \circ dW(t), \end{aligned}$$

where $dS_2$ denotes the stochastic differential of $S_2$ with respect to the *t* variable.

#### Proof

Choose an arbitrary pair $(q_a,p_a) \in T^*Q$ and define the particular solution $({\bar{q}}(\tau ), {\bar{p}}(\tau )) = F_{\tau ,t_a}(q_a,p_a)$. Form the function $S_2(q_a, {\bar{p}}(t),t)$ and consider its total stochastic differential^3^ ${\bar{d}}S_2(q_a, {\bar{p}}(t),t)$ with respect to time. On one hand, the rules of Stratonovich calculus imply

2.19 $$\begin{aligned} {\bar{d}}S_2(q_a, {\bar{p}}(t),t) = dS_2(q_a, {\bar{p}}(t),t) + \frac{\partial S_2}{\partial p}( q_a, {\bar{p}}(t),t )\circ d {\bar{p}}(t), \end{aligned}$$

where $dS_2$ denotes the partial stochastic differential of $S_2$ with respect to the *t* argument. On the other hand, integration by parts in (2.17) implies

2.20 $$\begin{aligned} {\bar{d}}S_2(q_a, {\bar{p}}(t),t) = {\bar{q}}(t)\circ d {\bar{p}}(t) + H({\bar{q}}(t),{\bar{p}}(t))\,dt + h({\bar{q}}(t),{\bar{p}}(t))\circ dW(t).\quad \end{aligned}$$

Comparing (2.19) and (2.20), and using (2.13), we obtain

2.21 $$\begin{aligned} d S_2(q_a, {\bar{p}}(t),t) = H\bigg (\frac{\partial S_2}{\partial p}\big (q_a, {\bar{p}}(t),t \big ), {\bar{p}}(t) \bigg )\,dt + h\bigg (\frac{\partial S_2}{\partial p}\big (q_a, {\bar{p}}(t),t\big ), {\bar{p}}(t) \bigg )\circ dW(t). \end{aligned}$$

This equation is satisfied along a particular path ${\bar{p}}(t)$, however, as in the discussion preceding Theorem 2.2, we can argue, slightly informally, that for almost all $\omega \in \varOmega$, and for *t* sufficiently close to $t_a$, one can find open neighborhoods $\mathscr {U}(\omega )\subset Q$ and $\mathscr {V}(\omega )\subset Q^*$ which can be connected by the flow $F_{t,t_a}$, i.e., given $q_a \in \mathscr {U}(\omega )$ and $p \in \mathscr {V}(\omega )$, there exists a pathwise unique solution $({\bar{q}}(\omega ,\tau ), {\bar{p}}(\omega ,\tau ))$ such that ${\bar{q}}(\omega ,t_a)=q_a$ and ${\bar{p}}(\omega ,t)=p$. With this assumption, (2.21) can be reformulated as the full-blown stochastic PDE (2.18). $\square$

**Remark** Similar stochastic Hamilton–Jacobi equations were introduced in [65, 66], where they were used for constructing stochastic symplectic integrators by considering series expansions of generating functions in terms of multiple Stratonovich integrals. This was a direct generalization of a similar technique for deterministic Hamiltonian systems (see [18]). In this work we explore the generalized Galerkin framework for constructing approximations of the generating function $S(q_a,p_b)$ in (2.13) by using its variational characterization (2.12). Our approach is a stochastic generalization of the techniques proposed in [33, 48] for deterministic Hamiltonian and Lagrangian systems.

### Stochastic Noether’s theorem

Let a Lie group *G* act on *Q* by the left action $\varPhi :G \times Q \longrightarrow Q$. The Lie group *G* then acts on *TQ* and $T^*Q$ by the tangent $\varPhi ^{TQ}:G \times TQ \longrightarrow TQ$ and cotangent $\varPhi ^{T^*Q}:G \times T^*Q \longrightarrow T^*Q$ lift actions, respectively, given in coordinates by the formulas (see [22, 38])

2.22 $$\begin{aligned} \varPhi ^{TQ}_g(q,{\dot{q}})&\equiv \varPhi ^{TQ}\big (g,(q,{\dot{q}})\big ) =\bigg ( \varPhi ^i_g(q),\frac{\partial \varPhi ^i_g}{\partial q^j}(q) {\dot{q}}^j \bigg ), \nonumber \\ \varPhi ^{T^*Q}_g(q,p)&\equiv \varPhi ^{T^*Q}\big (g,(q,p)\big ) =\bigg ( \varPhi ^i_g(q),p_j \frac{\partial \varPhi ^j_{g^{-1}}}{\partial q^i}\big (\varPhi _g(q)\big ) \bigg ), \end{aligned}$$

where $i,j=1,\ldots ,N$ and summation is implied over repeated indices. Let $\mathfrak {g}$ denote the Lie algebra of *G* and $\exp : \mathfrak {g} \longrightarrow G$ the exponential map (see [22, 38]). Each element $\xi \in \mathfrak {g}$ defines the infinitesimal generators $\xi _Q$, $\xi _{TQ}$, and $\xi _{T^*Q}$, which are vector fields on *Q*, *TQ*, and $T^*Q$, respectively, given by

2.23 $$\begin{aligned} \xi _Q(q) = \frac{d}{d \lambda } \bigg |_{\lambda =0} \varPhi _{\exp \lambda \xi }(q), \qquad \xi _{TQ}(q, {\dot{q}})&= \frac{d}{d \lambda } \bigg |_{\lambda =0} \varPhi ^{TQ}_{\exp \lambda \xi }(q,{\dot{q}}), \nonumber \\ \xi _{T^*Q}(q, p)&= \frac{d}{d \lambda } \bigg |_{\lambda =0} \varPhi ^{T^*Q}_{\exp \lambda \xi }(q,p). \end{aligned}$$

The momentum map $J: T^*Q \longrightarrow \mathfrak {g}^*$ associated with the action $\varPhi ^{T^*Q}$ is defined as the mapping such that for all $\xi \in \mathfrak {g}$ the function $J_\xi : T^*Q \ni (q,p) \longrightarrow \langle J(q,p),\xi \rangle \in {\mathbb {R}}$ is the Hamiltonian for the infinitesimal generator $\xi _{T^*Q}$, i.e.,

2.24 $$\begin{aligned} \xi ^q_{T^*Q} = \frac{\partial J_\xi }{\partial p}, \qquad \xi ^p_{T^*Q} = -\frac{\partial J_\xi }{\partial q}, \end{aligned}$$

where $\xi _{T^*Q}(q,p) = \big (q,p,\xi ^q_{T^*Q}(q,p),\xi ^p_{T^*Q}(q,p)\big )$. The momentum map *J* can be explicitly expressed as (see [22, 38])

2.25 $$\begin{aligned} J_\xi (q,p) = p\cdot \xi _Q(q). \end{aligned}$$

Noether’s theorem for deterministic Hamiltonian systems relates symmetries of the Hamiltonian to quantities preserved by the flow of the system. It turns out that this result carries over to the stochastic case, as well. A stochastic version of Noether’s theorem was proved in [6, 30]. For completeness, and for the benefit of the reader, below we state and provide a less involved proof of Noether’s theorem for stochastic Hamiltonian systems.

#### Theorem 2.3

(Stochastic Noether’s theorem) Suppose that the Hamiltonians $H:T^*Q \longrightarrow {\mathbb {R}}$ and $h:T^*Q \longrightarrow {\mathbb {R}}$ are invariant with respect to the cotangent lift action $\varPhi ^{T^*Q}:G \times T^*Q \longrightarrow T^*Q$ of the Lie group *G*, that is,

2.26 $$\begin{aligned} H \circ \varPhi ^{T^*Q}_g = H, \qquad h \circ \varPhi ^{T^*Q}_g = h, \end{aligned}$$

for all $g \in G$. Then the cotangent lift momentum map $J:T^*Q \longrightarrow \mathfrak {g}^*$ associated with $\varPhi ^{T^*Q}$ is almost surely preserved along the solutions of the stochastic Hamiltonian system (1.1).

#### Proof

Equation (2.26) implies that the Hamiltonians are infinitesimally invariant with respect to the action of *G*, that is, for all $\xi \in \mathfrak {g}$ we have

2.27 $$\begin{aligned} dH\cdot \xi _{T^*Q} = 0, \qquad dh\cdot \xi _{T^*Q} = 0, \end{aligned}$$

where *dH* and *dh* denote differentials with respect to the variables *q* and *p*. Let (*q*(*t*), *p*(*t*)) be a solution of (1.1) and consider the stochastic process $J_\xi (q(t),p(t))$, where $\xi \in \mathfrak {g}$ is arbitrary. Using the rules of Stratonovich calculus we can calculate the stochastic differential

2.28 $$\begin{aligned} dJ_\xi \big (q(t),p(t)\big )&=\frac{\partial J_\xi }{\partial q}(q(t),p(t))\circ dq + \frac{\partial J_\xi }{\partial p}(q(t),p(t))\circ dp \nonumber \\&=-\bigg ( \frac{\partial H}{\partial q} \xi ^q_{T^*Q} + \frac{\partial H}{\partial p} \xi ^p_{T^*Q} \bigg ) \,dt -\bigg ( \frac{\partial h}{\partial q} \xi ^q_{T^*Q} + \frac{\partial h}{\partial p} \xi ^p_{T^*Q} \bigg )\circ dW \nonumber \\&= -(dH\cdot \xi _{T^*Q})\,dt - (dh\cdot \xi _{T^*Q})\circ dW = 0, \end{aligned}$$

where we used (1.1), (2.24), and (2.27). Therefore, $J_\xi \big (q(t),p(t)\big ) = \text {const}$ almost surely for all $\xi \in \mathfrak {g}$, which completes the proof. $\square$

## Stochastic Galerkin Hamiltonian variational integrators

If the converse to Theorem 2.1 holds, then the generating function $S(q_a,p_b)$ defined in (2.12) could be equivalently characterized by

3.1 $$\begin{aligned} S(q_a,p_b) = \mathop {\hbox {ext}}\limits _{\begin{array}{c} (q,p)\in C([t_a,t_b])\\ q(t_a)=q_a,\,\, p(t_b)=p_b \end{array}} \mathscr {B}\big [q(\cdot ), p(\cdot ) \big ], \end{aligned}$$

where the extremum is taken pointwise in the probability space $\varOmega$. This characterization allows us to construct stochastic Galerkin variational integrators by choosing a finite dimensional subspace of $C([t_a,t_b])$ and a quadrature rule for approximating the integrals in the action functional $\mathscr {B}$. Galerkin variational integrators for deterministic systems were first introduced in [40], and further developed in [21, 32, 33, 47, 48]. In the remainder of the paper, we will generalize these ideas to the stochastic case.

### Construction of the integrator

Suppose we would like to solve (1.1) on the interval [0, *T*] with the initial conditions $(q_0,p_0)\in T^*Q$. Consider the discrete set of times $t_k = k\cdot \Delta t$ for $k=0,1,\ldots ,K$, where $\Delta t = T/K$ is the time step. In order to determine the discrete curve $\{(q_k,p_k)\}_{k=0,\ldots ,K}$ that approximates the exact solution of (1.1) at times $t_k$ we need to construct an approximation of the exact stochastic flow $F_{t_{k+1},t_k}$ on each interval $[t_k,t_{k+1}]$, so that $(q_{k+1},p_{k+1}) \approx F_{t_{k+1},t_k}(q_k,p_k)$. Let us consider the space $C^s([t_k,t_{k+1}])\subset C([t_k,t_{k+1}])$ defined as

3.2 $$\begin{aligned} C^s([t_k, t_{k+1}]) = \big \{ (q,p)\in C([t_k, t_{k+1}]) \, \big | \, q \text { is a polynomial of degree }s \big \}. \end{aligned}$$

For convenience, we will express *q*(*t*) in terms of Lagrange polynomials. Consider the control points $0=d_0<d_1<\cdots <d_s=1$ and let the corresponding Lagrange polynomials of degree *s* be denoted by $l_{\mu ,s}(\tau )$, that is, $l_{\mu ,s}(d_\nu )=\delta _{\mu \nu }$. A polynomial trajectory $q_d(t;q^\mu )$ can then be expressed as

3.3 $$\begin{aligned} q_d(t_k+\tau \Delta t; q^\mu ) = \sum _{\mu = 0}^s q^\mu l_{\mu ,s}(\tau ), \qquad {\dot{q}}_d(t_k+\tau \Delta t; q^\mu ) = \frac{1}{\Delta t}\sum _{\mu = 0}^s q^\mu \dot{l}_{\mu ,s}(\tau ), \end{aligned}$$

where $q^\nu = q_d(t_k+d_\nu \Delta t; q^\mu )$ for $\nu =0,\ldots ,s$ are the control values, ${\dot{q}}_d$ denotes the time derivative of $q_d$, and $\dot{l}_{\mu ,s}$ denotes the derivative of the Lagrange polynomial $l_{\mu ,s}$ with respect to its argument. The restriction of the action functional (2.4) to the space $C^s([t_k, t_{k+1}])$ takes the form

3.4 $$\begin{aligned} \mathscr {B}^s\big [q_d(\cdot ;q^\mu ),p(\cdot ) \big ]&= p(t_{k+1})q^s - \int _{t_k}^{t_{k+1}} \Big [ p(t){\dot{q}}_d(t) - H\big (q_d(t),p(t)\big ) \Big ] \,dt \nonumber \\&\quad +\, \int _{t_k}^{t_{k+1}} h\big (q_d(t),p(t)\big )\circ dW(t), \end{aligned}$$

since for differentiable functions $dq_d(t) = {\dot{q}}_d(t)\,dt$, where for brevity $q_d(t) \equiv q_d(t;q^\mu )$. Next we approximate the integrals in (3.4) using numerical quadrature rules $(\alpha _i, c_i)_{i=1}^r$ and $(\beta _i, c_i)_{i=1}^r$, where $0\le c_1<\cdots <c_r\le 1$ are the quadrature points, and $\alpha _i$, $\beta _i$ are the corresponding weights. At this point we only make a general assumption that for each *i* we have $\alpha _i \not = 0$ or $\beta _i \not =0$. More specific examples will be presented in Sect. 3.4. The approximate action functional takes the form

3.5 $$\begin{aligned}&\bar{\mathscr {B}}^s\big [q_d(\cdot ;q^\mu ),p(\cdot ) \big ] \nonumber \\&\quad =p(t_{k+1})q^s - \Delta t \sum _{i=1}^r \alpha _i \Big [ p(t_k+c_i\Delta t){\dot{q}}_d(t_k+c_i\Delta t) - H\big (q_d(t_k+c_i\Delta t),p(t_k+c_i\Delta t)\big ) \Big ] \nonumber \\&\qquad + \Delta W \sum _{i=1}^r \beta _i h\big (q_d(t_k+c_i\Delta t),p(t_k+c_i\Delta t)\big ), \end{aligned}$$

where $\Delta W = W(t_{k+1})-W(t_k)$ is the increment of the Wiener process over the considered time interval and is a Gaussian random variable with zero mean and variance $\Delta t$. The way of approximating the Stratonovich integral above was inspired by the ideas presented in [8, 12, 36, 43, 44]. Note that since we only used $\Delta W = \int _{t_k}^{t_{k+1}}dW(t)$ in the above approximation, we can in general expect mean-square convergence of order 1.0 only. To obtain mean-square convergence of higher order we would also need to include higher-order multiple Stratonovich integrals, e.g., to achieve convergence of order 1.5 we would need to include terms involving $\Delta Z = \int _{t_k}^{t_{k+1}}\int _{t_k}^{t}dW(\xi )\,dt$ (see [12, 43, 44]). We can now approximate the generating function $S(q_k,p_{k+1})$ with the discrete Hamiltonian function $H^+_d(q_k,p_{k+1})$ defined as

3.6 $$\begin{aligned} H^+_d(q_k,p_{k+1})&= \mathop {\hbox {ext}}_{ \begin{array}{c} q^1,\ldots ,q^s \in Q \\ P_1, \ldots , P_r \in Q^* \\ q^0 = q_k \end{array} } \bigg \{ p_{k+1}q^s - \Delta t \sum _{i=1}^r \alpha _i \Big [ P_i {\dot{q}}_d(t_k+c_i\Delta t) \nonumber \\&\quad - H\big (q_d(t_k+c_i\Delta t),P_i\big ) \Big ] \nonumber \\&\quad + \Delta W \sum _{i=1}^r \beta _i h\big (q_d(t_k+c_i\Delta t),P_i\big ) \bigg \}, \end{aligned}$$

where we denoted $P_i\equiv p(t_k+c_i\Delta t)$. The numerical scheme $(q_k,p_k)\longrightarrow (q_{k+1},p_{k+1})$ is now implicitly generated by $H^+_d(q_k,p_{k+1})$ as in (2.13):

3.7 $$\begin{aligned} q_{k+1} = D_2 H^+_d(q_k,p_{k+1}), \qquad p_k = D_1 H^+_d(q_k,p_{k+1}). \end{aligned}$$

Equations (3.6) and (3.7) can be written together as the following system:

3.8a $$\begin{aligned} -\,p_k&=\sum _{i=1}^r \alpha _i \left[ P_i {\dot{l}}_{0,s}(c_i) - \Delta t \frac{\partial H}{\partial q}\big (t_k+c_i\Delta t\big ) l_{0,s}(c_i) \right] \nonumber \\&\quad - \Delta W \sum _{i=1}^r \beta _i \frac{\partial h}{\partial q}\big (t_k+c_i\Delta t\big ) l_{0,s}(c_i), \end{aligned}$$

3.8b $$\begin{aligned} 0&=\sum _{i=1}^r \alpha _i \left[ P_i {\dot{l}}_{\mu ,s}(c_i) - \Delta t \frac{\partial H}{\partial q}\big (t_k+c_i\Delta t\big ) l_{\mu ,s}(c_i) \right] \nonumber \\&\quad - \Delta W \sum _{i=1}^r \beta _i \frac{\partial h}{\partial q}\big (t_k+c_i\Delta t\big ) l_{\mu ,s}(c_i), \end{aligned}$$

3.8c $$\begin{aligned} p_{k+1}&=\sum _{i=1}^r \alpha _i \left[ P_i {\dot{l}}_{s,s}(c_i) - \Delta t \frac{\partial H}{\partial q}\big (t_k+c_i\Delta t\big ) l_{s,s}(c_i) \right] \nonumber \\&\quad - \Delta W \sum _{i=1}^r \beta _i \frac{\partial h}{\partial q}\big (t_k+c_i\Delta t\big ) l_{s,s}(c_i), \end{aligned}$$

3.8d $$\begin{aligned} \alpha _i {\dot{q}}_d&(t_k+c_i\Delta t) = \alpha _i \frac{\partial H}{\partial p}\big (t_k+c_i\Delta t\big ) + \beta _i \frac{\Delta W}{\Delta t} \frac{\partial h}{\partial p}\big (t_k+c_i\Delta t\big ), \end{aligned}$$

3.8e $$\begin{aligned} q_{k+1}&=q^s, \end{aligned}$$

where $\mu =1,\ldots ,s-1$ in (3.8b), $i=1,\ldots , r$ in (3.8d), and for brevity we have introduced the notation

$$\begin{aligned} H(t_k+c_i\Delta t)\equiv H(q_d(t_k+c_i\Delta t),p(t_k+c_i\Delta t)) \qquad \text {(similarly for } h). \end{aligned}$$

Equation (3.8a) corresponds to the second equation in (3.7), Eqs. (3.8b), (3.8c) and (3.8d) correspond to extremizing (3.6) with respect to $q^1, \ldots , q^s$, and $P_1,\ldots , P_r$, respectively, and finally (3.8e) is the first equation in (3.7). Knowing $(q_k,p_k)$, the system (3.8) allows us to solve for $(q_{k+1},p_{k+1})$: we first simultaneously solve (3.8a), (3.8b) and (3.8d) [$(s+r)N$ equations] for $q^1, \ldots , q^s$ and $P_1,\ldots , P_r$ [$(s+r)N$ unknowns]; then $q_{k+1}=q^s$ and (3.8c) is an explicit update rule for $p_{k+1}$. When $h\equiv 0$, then (3.8) reduces to the deterministic Galerkin variational integrator discussed in [48]. Note that depending on the choice of the Hamiltonians and quadrature rules, the system (3.8) may be explicit, but in the general case it is implicit (see Sect. 3.4). One can use the Implicit Function Theorem to show that for sufficiently small $\Delta t$ and $|\Delta W|$ it will have a solution. However, since the increments $\Delta W$ are unbounded, for some values of $\Delta W$ solutions might not exist. To avoid problems with numerical implementations, if necessary, one can replace $\Delta W$ in (3.8) with the truncated random variable $\overline{\Delta W}$ defined as

3.9 $$\begin{aligned} \overline{\Delta W} = {\left\{ \begin{array}{ll} A, &{} \text {if }\Delta W > A,\\ \Delta W, &{} \text {if }|\Delta W| \le A, \\ -\,A, &{} \text {if }\Delta W < -\,A, \end{array}\right. } \end{aligned}$$

where $A>0$ is suitably chosen for the considered problem. See [14, 44] for more details regarding schemes with truncated random increments and their convergence. Alternatively, one could employ the techniques presented in, e.g., [51, 52, 67], where the unbounded increments $\Delta W$ have been replaced with discrete random variables.

Although the scheme (3.8) formally appears to be a straightforward generalization of its deterministic counterpart, it should be emphasized that the main difference lies in the fact that the increments $\Delta W$ are random variables such that $E(\Delta W^2)=\Delta t$, which makes the convergence analysis significantly more challenging than in the deterministic case. The main difficulty is in the choice of the parameters *s*, *r*, $\alpha _i$, $\beta _i$, $c_i$, so that the resulting numerical scheme converges in some sense to the solutions of (1.1). The number of parameters and order conditions grows rapidly, when terms approximating multiple Stratonovich integrals are added (see Sect. 3.6 and [10–12, 14]). In Sects. 3.2 and 3.3 we study the geometric properties of the family of schemes described by (3.8), whereas in Sects. 3.4 and 3.5 we provide concrete choices of the coefficients that lead to convergent methods.

### Properties of stochastic Galerkin variational integrators

The key features of variational integrators are their symplecticity and exact preservation of the discrete counterparts of conserved quantities (momentum maps) related to the symmetries of the Lagrangian or Hamiltonian (see [40]). These properties carry over to the stochastic case, as was first demonstrated in [8] for Lagrangian systems. In what follows, we will show that the stochastic Galerkin Hamiltonian variational integrators constructed in Sect. 3.1 also possess these properties.

#### Theorem 3.1

(Symplecticity of the discrete flow) Let $F^+_{t_{k+1},t_k}:\varOmega \times T^*Q \longrightarrow T^*Q$ be the dicrete stochastic flow implicitly defined by the discrete Hamiltonian $H^+_d$ as in (3.7). Then $F^+_{t_{k+1},t_k}$ is almost surely symplectic, that is,

3.10 $$\begin{aligned} (F^+_{t_{k+1},t_k})^* \varOmega _{T^*Q} = \varOmega _{T^*Q}, \end{aligned}$$

where $\varOmega _{T^*Q} = \sum _{i=1}^N dq^i \wedge dp^i$ is the canonical symplectic form on $T^*Q$.

#### Proof

The proof follows immediately by observing that (see [33])

3.11 $$\begin{aligned} 0 \!=\! ddH^+(q_k,p_{k+1}) \!=\! \sum _{i=1}^N dq^i_{k+1} \wedge dp^i_{k+1} - \sum _{i=1}^N dq^i_{k} \wedge dp^i_{k} \!=\! (F^+_{t_{k+1},t_k})^* \varOmega _{T^*Q} - \varOmega _{T^*Q}, \end{aligned}$$

where *d* in the above formula denotes the differential operator with respect to the variables *q* and *p* and is understood in the mean-square sense. The result holds almost surely, because Eq. (3.7) holds almost surely. $\square$

The discrete counterpart of stochastic Noether’s theorem readily generalizes from the corresponding theorem in the deterministic case.

#### Theorem 3.2

(Discrete stochastic Noether’s theorem) Let $\varPhi ^{T^*Q}$ be the cotangent lift action of the action $\varPhi$ of the Lie group *G* on the configuration space *Q*. If the generalized discrete stochastic Lagrangian $R_d(q_k, p_{k+1}) = p_{k+1}q_{k+1} - H^+_d(q_k,p_{k+1})$, where $q_{k+1}=D_2H^+_d(q_k,p_{k+1})$, is invariant under the action of *G*, that is,

3.12 $$\begin{aligned} R_d \big ( \varPhi _g(q_k), \pi _{Q^*}\circ \varPhi ^{T^*Q}_g(q_{k+1},p_{k+1}) \big ) = R_d(q_k, p_{k+1}), \quad \text {for all }g\in G, \end{aligned}$$

where $\pi _{Q^*}: Q \times Q^* \longrightarrow Q^*$ is the projection onto $Q^*$, then the cotangent lift momentum map *J* associated with $\varPhi ^{T^*Q}$ is almost surely preserved, i.e., a.s. $J(q_{k+1},p_{k+1}) = J (q_{k},p_{k})$.

#### Proof

See the proof of Theorem 4 in [33]. In our case the result holds almost surely, because Eq. (3.7) holds almost surely. $\square$

For applications, it is useful to know under what conditions the discrete Hamiltonian (3.6) inherits the symmetry properties of the Hamiltonians *H* and *h*. Not unexpectedly, this depends on the behavior of the interpolating polynomial (3.3) under the group action. We say that the polynomial $q_d(t; q^\mu )$ is equivariant with respect to *G* if for all $g \in G$ we have

3.13 $$\begin{aligned} \varPhi ^{TQ}_g \Big ( q_d(t; q^\mu ), {\dot{q}}_d(t; q^\mu ) \Big ) = \Big ( q_d\big (t; \varPhi _g(q^\mu )\big ), {\dot{q}}_d\big (t; \varPhi _g(q^\mu )\big ) \Big ). \end{aligned}$$

#### Theorem 3.3

Suppose that the Hamiltonians $H:T^*Q \longrightarrow {\mathbb {R}}$ and $h:T^*Q \longrightarrow {\mathbb {R}}$ are invariant with respect to the cotangent lift action $\varPhi ^{T^*Q}:G \times T^*Q \longrightarrow T^*Q$ of the Lie group *G*, that is,

3.14 $$\begin{aligned} H \circ \varPhi ^{T^*Q}_g = H, \qquad h \circ \varPhi ^{T^*Q}_g = h, \end{aligned}$$

for all $g \in G$, and suppose the interpolating polynomial $q_d(t; q^\mu )$ is equivariant with respect to *G*. Then the generalized discrete stochastic Lagrangian $R_d(q_k, p_{k+1}) = p_{k+1}q_{k+1} - H^+_d(q_k,p_{k+1})$ corresponding to the discrete Hamiltonian (3.6), where $q_{k+1}=D_2H^+_d(q_k,p_{k+1})$, is invariant with respect to the action of *G*.

#### Proof

The proof is similar to the proofs of Lemma 3 in [33] and Theorem 3 in [48]. Let us, however, carefully examine the actions of *G* on *Q*, *TQ*, and $T^*Q$. Let $q_k \in Q$ and $p_{k+1} \in Q^*$, and let $q_{k+1}=D_2H^+_d(q_k,p_{k+1})$. First, note that for the stochastic discrete Hamiltonian (3.6), we have

3.15 $$\begin{aligned} R(q_k, p_{k+1})&= \mathop {\hbox {ext}}_{ \begin{array}{c} q^1,\ldots ,q^s \in Q \\ P_1, \ldots , P_r \in Q^* \\ q^0 = q_k \end{array} } \left\{ \Delta t \sum _{i=1}^r \alpha _i \Big [ P_i {\dot{q}}_d(t_k+c_i\Delta t; q^\mu ) - H\big (q_d(t_k+c_i\Delta t; q^\mu ),P_i\big ) \Big ]\right. \nonumber \\&\quad \left. - \Delta W \sum _{i=1}^r \beta _i h\big (q_d(t_k+c_i\Delta t; q^\mu ),P_i\big ) \right\} , \end{aligned}$$

where we used (3.8e). Consider $\tilde{q}_k = \varPhi _g(q_k)$ and $(\tilde{q}_{k+1}, \tilde{p}_{k+1}) = \varPhi ^{T^*Q}_g(q_{k+1},p_{k+1})$ for $g \in G$, and calculate (3.15) for the transformed values $\tilde{q}_k$ and $\tilde{p}_{k+1}$:

3.16 $$\begin{aligned} R(\tilde{q}_k, \tilde{p}_{k+1})&= \mathop {\hbox {ext}}_{ \begin{array}{c} \tilde{q}^1,\ldots ,\tilde{q}^s \in Q \\ \tilde{P}_1, \ldots , \tilde{P}_r \in Q^* \\ \tilde{q}^0 = \tilde{q}_k \end{array} } \left\{ \Delta t \sum _{i=1}^r \alpha _i \Big [ \tilde{P}_i {\dot{q}}_d(t_k+c_i\Delta t; \tilde{q}^\mu ) - H\big (q_d(t_k+c_i\Delta t; \tilde{q}^\mu ),\tilde{P}_i\big ) \Big ] \right. \nonumber \\&\quad \left. - \Delta W \sum _{i=1}^r \beta _i h\big (q_d(t_k+c_i\Delta t; \tilde{q}^\mu ),\tilde{P}_i\big ) \right\} . \end{aligned}$$

Let us perform a change of variables with respect to which we extremize. First, define $q^\mu = \varPhi _{g^{-1}}(\tilde{q}^\mu )$, so that $\tilde{q}^\mu = \varPhi _{g}(q^\mu )$ for $\mu =0,\ldots ,s$. From (3.13) we have $q_d(t_k+c_i\Delta t; \tilde{q}^\mu ) = \varPhi _g ( q_d(t_k+c_i\Delta t; q^\mu ))$, which we use to define $P_i$ by $\big (q_d(t_k+c_i\Delta t; \tilde{q}^\mu ),\tilde{P}_i\big ) = \varPhi ^{T*Q}_g\big (q_d(t_k+c_i\Delta t; q^\mu ),P_i\big )$ for $i=1,\ldots ,r$. Since $\varPhi _g$ and $\varPhi ^{T*Q}_g$ are bijective, extremization with respect to $q^\mu$ and $P_i$ is equivalent to extremization with respect to $\tilde{q}^\mu$ and $\tilde{P}_i$, and $\tilde{q}^0 = \tilde{q}_k$ implies $q^0 = q_k$. Moreover, from (3.13) and (2.22) we have that $\tilde{P}_i {\dot{q}}_d(t_k+c_i\Delta t; \tilde{q}^\mu ) = P_i {\dot{q}}_d(t_k+c_i\Delta t; q^\mu )$. Finally, the invariance of the Hamiltonians implies

3.17 $$\begin{aligned} R(\tilde{q}_k, \tilde{p}_{k+1})&= \mathop {\hbox {ext}}_{ \begin{array}{c} q^1,\ldots ,q^s \in Q \\ P_1, \ldots , P_r \in Q^* \\ q^0 = q_k \end{array} } \left\{ \Delta t \sum _{i=1}^r \alpha _i \Big [ P_i {\dot{q}}_d(t_k+c_i\Delta t; q^\mu ) - H\big (q_d(t_k+c_i\Delta t; q^\mu ),P_i\big ) \Big ]\right. \nonumber \\&\quad \left. - \Delta W \sum _{i=1}^r \beta _i h\big (q_d(t_k+c_i\Delta t; q^\mu ),P_i\big ) \right\} = R(q_k, p_{k+1}), \end{aligned}$$

which completes the proof. $\square$

**Remark** One can easily verify that the interpolating polynomial (3.3) is in particular equivariant with respect to linear actions (translations, rotations, etc.), therefore the stochastic Galerkin variational integrator (3.8) preserves quadratic momentum maps (such as linear and angular momentum) related to linear symmetries of the Hamiltonians *H* and *h*.

### Stochastic symplectic partitioned Runge–Kutta methods

A general class of stochastic Runge–Kutta methods for Stratonovich ordinary differential equations was introduced and analyzed in [10–12]. These ideas were later used by Ma et al. [35] and Ma and Ding [36] to construct symplectic Runge–Kutta methods for stochastic Hamiltonian systems. An *s*-stage stochastic symplectic partitioned Runge–Kutta method for (1.1) is defined in [36] by the following system:

3.18a $$\begin{aligned} Q_i&= q_k + \Delta t \sum _{j=1}^s a_{ij} \frac{\partial H}{\partial p}(Q_j,P_j) + \Delta W \sum _{j=1}^s b_{ij} \frac{\partial h}{\partial p}(Q_j,P_j), \quad i=1,\ldots ,s, \end{aligned}$$

3.18b $$\begin{aligned} P_i&= p_k - \Delta t \sum _{j=1}^s {\bar{a}}_{ij} \frac{\partial H}{\partial q}(Q_j,P_j) - \Delta W \sum _{j=1}^s {\bar{b}}_{ij} \frac{\partial h}{\partial q}(Q_j,P_j), \quad i=1,\ldots ,s, \end{aligned}$$

3.18c $$\begin{aligned} q_{k+1}&= q_k + \Delta t \sum _{i=1}^s \alpha _i \frac{\partial H}{\partial p}(Q_i,P_i) + \Delta W \sum _{i=1}^s \beta _i \frac{\partial h}{\partial p}(Q_i,P_i), \end{aligned}$$

3.18d $$\begin{aligned} p_{k+1}&= p_k - \Delta t \sum _{i=1}^s \alpha _i \frac{\partial H}{\partial q}(Q_i,P_i) - \Delta W \sum _{i=1}^s \beta _i \frac{\partial h}{\partial q}(Q_i,P_i), \end{aligned}$$

where $Q_i$ and $P_i$ for $i=1,\ldots ,s$ are the position and momentum internal stages, respectively, and the coefficients of the method $a_{ij}$, ${\bar{a}}_{ij}$, $b_{ij}$, ${\bar{b}}_{ij}$, $\alpha _i$, $\beta _i$ satisfy the symplectic conditions

3.19a $$\begin{aligned} \alpha _i {\bar{a}}_{ij} + \alpha _j a_{ji}&= \alpha _i \alpha _j, \end{aligned}$$

3.19b $$\begin{aligned} \beta _i {\bar{a}}_{ij} + \alpha _j b_{ji}&= \beta _i \alpha _j, \end{aligned}$$

3.19c $$\begin{aligned} \alpha _i {\bar{b}}_{ij} + \beta _j a_{ji}&= \alpha _i \beta _j, \end{aligned}$$

3.19d $$\begin{aligned} \beta _i {\bar{b}}_{ij} + \beta _j b_{ji}&= \beta _i \beta _j, \end{aligned}$$

for $i,j=1,\ldots ,s$. We now prove that in the special case when $r=s$, the stochastic Galerkin variational integrator (3.8) is equivalent to the stochastic symplectic partitioned Runge–Kutta method (3.18).

#### Theorem 3.4

Let $r=s$ and let ${\bar{l}}_{i,s-1}(\tau )$ for $i=1,\ldots , s$ denote the Lagrange polynomials of degree $s-1$ associated with the quadrature points $0\le c_1< \cdots <c_s \le 1$. Moreover, let the weights $\alpha _i$ be given by

3.20 $$\begin{aligned} \alpha _i = \int _0^1 {\bar{l}}_{i,s-1}(\tau )\,d\tau , \end{aligned}$$

and assume $\alpha _i \not = 0$ for $i=1,\ldots , s$. Then the stochastic Galerkin Hamiltonian variational integrator (3.8) is equivalent to the stochastic partitioned Runge–Kutta method (3.18) with the coefficients

3.21a $$\begin{aligned} a_{ij}&=\int _0^{c_i} {\bar{l}}_{j,s-1}(\tau )\,d\tau , \end{aligned}$$

3.21b $$\begin{aligned} {\bar{a}}_{ij}&= \frac{\alpha _j(\alpha _i-a_{ji})}{\alpha _i}, \end{aligned}$$

3.21c $$\begin{aligned} b_{ij}&= \frac{\beta _j a_{ij}}{\alpha _j}, \end{aligned}$$

3.21d $$\begin{aligned} {\bar{b}}_{ij}&= \frac{\beta _j(\alpha _i-a_{ji})}{\alpha _i}, \end{aligned}$$

for $$i,j=1,\ldots ,s$$.

#### Proof

The proof follows the main steps of the proof of Theorem 2.6.2 in [40]. The time derivative ${\dot{q}}_d$ is a polynomial of degree $s-1$. Therefore, it can be uniquely expressed in terms of the Lagrange polynomials ${\bar{l}}_{j,s-1}(\tau )$ as

3.22 $$\begin{aligned} {\dot{q}}_d(t_k+\tau \Delta t) = \sum _{j=1}^s {\dot{q}}_d(t_k+c_j \Delta t) {\bar{l}}_{j,s-1}(\tau ). \end{aligned}$$

Upon integrating with respect to time, we find

3.23 $$\begin{aligned} q_d(t_k+\tau \Delta t) = q_k + \Delta t \sum _{j=1}^s {\dot{q}}_d(t_k+c_j \Delta t) \int _0^\tau {\bar{l}}_{j,s-1}(\xi )\,d\xi , \end{aligned}$$

where we have used $q^0=q_k$. For $\tau =1$ this gives

3.24 $$\begin{aligned} q_{k+1} = q_k + \Delta t \sum _{j=1}^s \alpha _j {\dot{q}}_d(t_k+c_j \Delta t), \end{aligned}$$

where we have used $q^s = q_{k+1}$ and (3.20). Define the internal stages $Q_j\equiv q_d(t_k+c_j \Delta t)$. Then, upon using (3.8d), Eq. (3.24) becomes (3.18c). For $\tau =c_i$ Eq. (3.23) gives

3.25 $$\begin{aligned} Q_i = q_k + \Delta t \sum _{j=1}^s a_{ij} {\dot{q}}_d(t_k+c_j \Delta t), \end{aligned}$$

where $a_{ij}$ is defined by (3.21a). Upon substituting (3.8d), Eq. (3.25) becomes (3.18a), where $b_{ij}$ is defined by (3.21c). Next, sum Eqs. (3.8a), (3.8b), and (3.8c). Noting that $\sum _{\mu =0}^s l_{\mu ,s}(\tau ) = 1$, this gives Eq. (3.18d). Finally, we note that for each $i=1,\ldots ,s$ we have a unique decomposition

3.26 $$\begin{aligned} \int _0^\tau {\bar{l}}_{i,s-1}(\xi )\,d\xi - \alpha _i = \sum _{\mu =0}^s m_{i \mu } l_{\mu ,s}(\tau ), \end{aligned}$$

since the left-hand side is a polynomial of degree *s*, and therefore it can be uniquely expressed as a linear combination of the Lagrange polynomials $l_{\mu ,s}(\tau )$ with the coefficients $m_{i\mu }$. Evaluating this identity at $\tau =d_0=0$, $\tau =d_s=1$, and differentiating it with respect to $\tau$ yield the following three equations, respectively,

3.27 $$\begin{aligned} -\,\alpha _i&= \sum _{\mu =0}^s m_{i \mu } l_{\mu ,s}(0) = m_{i0}, \nonumber \\ 0&= \sum _{\mu =0}^s m_{i \mu } l_{\mu ,s}(1) = m_{is}, \nonumber \\ {\bar{l}}_{i,s-1}(\tau )&= \sum _{\mu =0}^s m_{i \mu } {\dot{l}}_{\mu ,s}(\tau ). \end{aligned}$$

We form a linear combination of Eqs. (3.8a), (3.8b) and (3.8c) with the coefficients $m_{j0}$, $m_{j\mu }$, and $m_{js}$, respectively. By using the identities (3.27) and rearranging the terms, we obtain (3.18d), where ${\bar{a}}_{ij}$ and ${\bar{b}}_{ij}$ are defined by (3.21b) and (3.21d), respectively. One can easily verify that the coefficients (3.21) satisfy the conditions (3.19). $\square$

### Examples

In the construction of the integrator (3.8) we may choose the degree *s* of the approximating polynomials and the quadrature rules $(\alpha _i, c_i)_{i=1}^r$ and $(\beta _i, c_i)_{i=1}^r$. In the deterministic case, the higher the degree of the polynomials and the higher the order of the quadrature rule, then the higher the order of convergence of the resulting integrator (see [48]). In our case, however, as explained in Sect. 3.1, we cannot in general achieve mean-square order of convergence higher than 1.0, because we only used $\Delta W$ in (3.5). Since the system (3.8) requires solving $(s+r)N$ equations for $(s+r)N$ variables, from the computational point of view it makes sense to only consider methods with low values of *s* and *r*. In this work we focus on the following classical numerical integration formulas (see [18–20]):

- Gauss–Legendre quadratures (Gau): midpoint rule ($r=1$), etc.
- Lobatto quadratures (Lob): trapezoidal rule ($r=2$), Simpson’s rule ($r=3$), etc.
- Open trapezoidal rule (Otr; $r=2$)
- Milne’s rule (Mil; $r=3$)
- Rectangle rule (Rec; $r=1$)—only in the case when $h=h(q)$.

In [48] notation *PsNrQu* was proposed to denote a Galerkin variational integrator based on polynomials of degree *s* and a quadrature rule of order *u* with *r* quadrature points. We adopt a similar notation, keeping in mind that *u* denotes the *classical* order of the used quadrature rule—when the rule is applied to a stochastic integral, as in (3.5), its classical order is not attained in general. We also use a three-letter code to identify which integration formula is used. For example, *P*2*N*2*Q*4*Gau* denotes the integrator defined by (3.8) using polynomials of degree 2 and the Gauss–Legendre quadrature formula of classical order 4 with 2 quadrature points for both the Lebesgue and Stratonovich integrals in (3.5). If two different quadrature rules are used, we first write the rule applied to the Lebesgue integral, followed by the rule applied to the Stratonovich integral, e.g., *P*1*N*1*Q*2*GauN*2*Q*2*Lob*. Below we give several examples of integrators obtained by using polynomials of degree $s=1,2$ and the quadrature rules listed above.

#### General Hamiltonian function *h*(*q*, *p*)

For a general Hamiltonian $h=h(q,p)$, Eq. (3.8d), which represents the discretization of the Legendre transform, needs to contain both $\partial H / \partial p$ and $\partial h / \partial p$ terms to correctly approximate the continuous system. Therefore, we only consider methods with $\alpha _i=\beta _i\not = 0$ for all $i=1,\ldots ,r$. A few examples of interest are listed below.

1. *P*1*N*1*Q*2*Gau* (*Stochastic midpoint method*)
  Using the midpoint rule ($r=1$, $c_1=1/2$, $\alpha _1=\beta _1=1$) together with polynomials of degree $s=1$ gives a stochastic Runge–Kutta method (3.18) with $a_{11}={\bar{a}}_{11}=b_{11}={\bar{b}}_{11}=1/2$. Noting that $Q_1=(q_k+q_{k+1})/2$ and $P_1=(p_k+p_{k+1})/2$, this method can be written as
  3.28 $$\begin{aligned} q_{k+1}&= q_k + \frac{\partial H}{\partial p} \bigg (\frac{q_k+q_{k+1}}{2},\frac{p_k+p_{k+1}}{2} \bigg )\Delta t + \frac{\partial h}{\partial p} \bigg (\frac{q_k+q_{k+1}}{2},\frac{p_k+p_{k+1}}{2} \bigg )\Delta W, \nonumber \\ p_{k+1}&= p_k - \frac{\partial H}{\partial q} \bigg (\frac{q_k+q_{k+1}}{2},\frac{p_k+p_{k+1}}{2} \bigg )\Delta t - \frac{\partial h}{\partial q} \bigg (\frac{q_k+q_{k+1}}{2},\frac{p_k+p_{k+1}}{2} \bigg )\Delta W. \end{aligned}$$
  The stochastic midpoint method was considered in [36, 44]. It is an implicit method and in general one has to solve 2*N* equations for 2*N* unknowns. However, if the Hamiltonians are separable, that is, $H(q,p)=T_0(p)+U_0(q)$ and $h(q,p)=T_1(p)+U_1(q)$, then $p_{k+1}$ from the second equation can be substituted into the first one. In that case only *N* nonlinear equations have to be solved for $q_{k+1}$.
2. *P*2*N*2*Q*2*Lob* (*Stochastic Störmer–Verlet method*)
  If the trapezoidal rule ($r=2$, $c_1=0$, $c_2=1$, $\alpha _1=\beta _1=1/2$, $\alpha _2=\beta _2=1/2$) is used with polynomials of degree $s=2$, we obtain another partitioned Runge–Kutta method (3.18) with $a_{11}=a_{12}=0$, $a_{21}=a_{22}=1/2$, ${\bar{a}}_{11}={\bar{a}}_{21}=1/2$, ${\bar{a}}_{12}={\bar{a}}_{22}=0$, $(b_{ij})=(a_{ij})$, $({\bar{b}}_{ij})=({\bar{a}}_{ij})$. Noting that $Q_1=q_k$, $Q_2=q_{k+1}$, and $P_1=P_2$, this method can be more efficiently written as
  3.29 $$\begin{aligned} P_1&= p_k - \frac{1}{2} \frac{\partial H}{\partial q} \big (q_k, P_1 \big )\Delta t - \frac{1}{2} \frac{\partial h}{\partial q} \big (q_k, P_1 \big )\Delta W, \nonumber \\ q_{k+1}&= q_k + \frac{1}{2} \frac{\partial H}{\partial p} \big (q_k, P_1 \big )\Delta t + \frac{1}{2} \frac{\partial H}{\partial p} \big (q_{k+1}, P_1 \big )\Delta t \nonumber \\&\quad + \frac{1}{2} \frac{\partial h}{\partial p} \big (q_k, P_1 \big )\Delta W + \frac{1}{2} \frac{\partial h}{\partial p} \big (q_{k+1}, P_1 \big )\Delta W, \nonumber \\ p_{k+1}&= P_1 - \frac{1}{2} \frac{\partial H}{\partial q} \big (q_{k+1}, P_1 \big )\Delta t - \frac{1}{2} \frac{\partial h}{\partial q} \big (q_{k+1}, P_1 \big )\Delta W. \end{aligned}$$
  This method is a stochastic generalization of the Störmer–Verlet method (see [18]) and was considered in [36]. It is particularly efficient, because the first equation can be solved separately from the second one, and the last equation is an explicit update. Moreover, if the Hamiltonians are separable, this method becomes fully explicit.
3. *P*1*N*2*Q*2*Lob* (*Stochastic trapezoidal method*)
  This integrator is based on polynomials of degree $s=1$ with control points $d_0=0$, $d_1=1$, and the trapezoidal rule. Equations (3.8) take the form
  3.30 $$\begin{aligned} p_k&= \frac{1}{2}(P_1+P_2) + \frac{1}{2} \frac{\partial H}{\partial q} \big (q_k, P_1 \big )\Delta t + \frac{1}{2} \frac{\partial h}{\partial q} \big (q_k, P_1 \big )\Delta W, \nonumber \\ p_{k+1}&= \frac{1}{2}(P_1+P_2) - \frac{1}{2} \frac{\partial H}{\partial q} \big (q_{k+1}, P_2 \big )\Delta t - \frac{1}{2} \frac{\partial h}{\partial q} \big (q_{k+1}, P_2 \big )\Delta W, \nonumber \\ q_{k+1}&= q_k + \frac{\partial H}{\partial p} \big (q_k,P_1 \big )\Delta t + \frac{\partial h}{\partial p} \big (q_k,P_1 \big )\Delta W, \nonumber \\ q_{k+1}&= q_k + \frac{\partial H}{\partial p} \big (q_{k+1},P_2 \big )\Delta t + \frac{\partial h}{\partial p} \big (q_{k+1},P_2 \big )\Delta W. \end{aligned}$$
  This integrator is a stochastic generalization of the trapezoidal method for deterministic systems (see [40]). One may easily verify that if the Hamiltonians are separable, that is, $H(q,p)=T_0(p)+U_0(q)$ and $h(q,p)=T_1(p)+U_1(q)$, then $P_1=P_2$ and (3.30) is equivalent to the Störmer–Verlet method (3.29) and is fully explicit.
4. *P*1*N*3*Q*4*Lob*
  If we use Simpson’s rule ($r=3$, $c_1=0$, $c_2=1/2$, $c_3=1$, $\alpha _1=1/6$, $\alpha _2=2/3$, $\alpha _3=1/6$, $\beta _i=\alpha _i$), the resulting integrator (3.8) requires solving simultaneously 4*N* nonlinear equations, so it is computationally expensive in general. However, if the Hamiltonians *H* and *h* are separable, then (3.8d) implies $P_1=P_2=P_3$, and the integrator can be rewritten as
  3.31 $$\begin{aligned} q_{k+1}&= q_k + \frac{\partial T_0}{\partial p} \big (P_1 \big )\Delta t + \frac{\partial T_1}{\partial p} \big (P_1 \big )\Delta W, \nonumber \\ p_{k+1}&= P_1 - \frac{1}{3} \frac{\partial U_0}{\partial q} \bigg (\frac{q_k+q_{k+1}}{2}\bigg )\Delta t - \frac{1}{6} \frac{\partial U_0}{\partial q} \big (q_{k+1}\big )\Delta t \nonumber \\&\quad - \frac{1}{3} \frac{\partial U_1}{\partial q} \bigg (\frac{q_k+q_{k+1}}{2}\bigg )\Delta W - \frac{1}{6} \frac{\partial U_1}{\partial q} \big (q_{k+1}\big )\Delta W, \end{aligned}$$
  where
  3.32 $$\begin{aligned} P_1&= p_k - \frac{1}{6} \frac{\partial U_0}{\partial q} \big (q_k\big )\Delta t - \frac{1}{3} \frac{\partial U_0}{\partial q} \bigg (\frac{q_k+q_{k+1}}{2}\bigg )\Delta t \nonumber \\&\quad - \frac{1}{6} \frac{\partial U_1}{\partial q} \big (q_k\big )\Delta W - \frac{1}{3} \frac{\partial U_1}{\partial q} \bigg (\frac{q_k+q_{k+1}}{2}\bigg )\Delta W, \end{aligned}$$
  and $H(q,p)=T_0(p)+U_0(q)$ and $h(q,p)=T_1(p)+U_1(q)$. In this case only the first equation needs to be solved for $q_{k+1}$, and then the second equation is an explicit update.
5. *P*1*N*2*Q*2*Otr*
  Like the method (3.30), this integrator is based on polynomials of degree $s=1$ with control points $d_0=0$, $d_1=1$, but uses the open trapezoidal rule ($r=2$, $c_1=1/3$, $c_2=2/3$, $\alpha _1=1/2$, $\alpha _2=1/2$, $\beta _i=\alpha _i$). Equations (3.8) take the form
  3.33 $$\begin{aligned} p_k&= \frac{1}{2}(P_1+P_2) + \frac{1}{3} \frac{\partial H}{\partial q} \bigg (\frac{q_{k+1}+2q_k}{3}, P_1 \bigg )\Delta t + \frac{1}{6} \frac{\partial H}{\partial q} \bigg (\frac{2q_{k+1}+q_k}{3}, P_2 \bigg )\Delta t \nonumber \\&\quad + \frac{1}{3} \frac{\partial h}{\partial q} \bigg (\frac{q_{k+1}+2q_k}{3}, P_1 \bigg )\Delta W + \frac{1}{6} \frac{\partial h}{\partial q} \bigg (\frac{2q_{k+1}+q_k}{3}, P_2 \bigg )\Delta W, \nonumber \\ p_{k+1}&= \frac{1}{2}(P_1+P_2) - \frac{1}{6} \frac{\partial H}{\partial q} \bigg (\frac{q_{k+1}+2q_k}{3}, P_1 \bigg )\Delta t - \frac{1}{3} \frac{\partial H}{\partial q} \bigg (\frac{2q_{k+1}+q_k}{3}, P_2 \bigg )\Delta t \nonumber \\&\quad - \frac{1}{6} \frac{\partial h}{\partial q} \bigg (\frac{q_{k+1}+2q_k}{3}, P_1 \bigg )\Delta W - \frac{1}{3} \frac{\partial h}{\partial q} \bigg (\frac{2q_{k+1}+q_k}{3}, P_2 \bigg )\Delta W, \nonumber \\ q_{k+1}&= q_k + \frac{\partial H}{\partial p} \bigg (\frac{q_{k+1}+2q_k}{3}, P_1 \bigg )\Delta t + \frac{\partial h}{\partial p} \bigg (\frac{q_{k+1}+2q_k}{3}, P_1 \bigg )\Delta W, \nonumber \\ q_{k+1}&= q_k + \frac{\partial H}{\partial p} \bigg (\frac{2q_{k+1}+q_k}{3}, P_2 \bigg )\Delta t + \frac{\partial h}{\partial p} \bigg (\frac{2q_{k+1}+q_k}{3}, P_2 \bigg )\Delta W. \end{aligned}$$
  In general one has to solve the first, third, and fourth equation simultaneously (3*N* equations for 3*N* variables). In case of separable Hamiltonians we have $P_1=P_2$ and one only needs to solve *N* nonlinear equations: $P_1$ can be explicitly calculated from the first equation and substituted into the third one, and the resulting nonlinear equation then has to be solved for $q_{k+1}$.
6. *P*2*N*2*Q*2*Otr*
  If the open trapezoidal rule is used with polynomials of degree $s=2$, we obtain yet another partitioned Runge–Kutta method (3.18) with $a_{11}={\bar{a}}_{22}=1/2$, $a_{12}={\bar{a}}_{12}=-1/6$, $a_{21}={\bar{a}}_{21}=2/3$, $a_{22}={\bar{a}}_{11}=0$, $(b_{ij})=(a_{ij})$, $({\bar{b}}_{ij})=({\bar{a}}_{ij})$. Inspecting Eq. (3.18) we see that, for example, $Q_2$ is explicitly given in terms of $Q_1$ and $P_1$, therefore one only needs to solve 3*N* equations for the 3*N* variables $Q_1$, $P_1$, $P_2$, and the remaining equations are explicit updates. This method further simplifies for separable Hamiltonians *H* and *h*: $Q_1$ and $Q_2$ are now explicitly given in terms of $P_1$ and $P_2$, and the nonlinear equation for $P_1$ can be solved separately from the nonlinear equation for $P_2$.
7. *P*1*N*3*Q*4*Mil*
  A method similar to (3.31) is obtained if we use Milne’s rule ($r=3$, $c_1=1/4$, $c_2=1/2$, $c_3=3/4$, $\alpha _1=2/3$, $\alpha _2=-1/3$, $\alpha _3=2/3$, $\beta _i=\alpha _i$) instead of Simpson’s rule. The resulting integrator is also computationally expensive in general, but if the Hamiltonians *H* and *h* are separable, then (3.8d) implies $P_1=P_2=P_3$, and the integrator can be rewritten as
  3.34 $$\begin{aligned} q_{k+1}&= q_k + \frac{\partial T_0}{\partial p} \big (P_1 \big )\Delta t + \frac{\partial T_1}{\partial p} \big (P_1 \big )\Delta W, \nonumber \\ p_{k+1}&= p_k - \frac{2}{3} \frac{\partial U_0}{\partial q} \bigg (\frac{3 q_k+q_{k+1}}{4}\bigg )\Delta t + \frac{1}{3} \frac{\partial U_0}{\partial q} \bigg (\frac{q_k+q_{k+1}}{2}\bigg )\Delta t \nonumber \\&\quad - \frac{2}{3} \frac{\partial U_0}{\partial q} \bigg (\frac{q_k+3 q_{k+1}}{4}\bigg )\Delta t - \frac{2}{3} \frac{\partial U_1}{\partial q} \bigg (\frac{3 q_k+q_{k+1}}{4}\bigg )\Delta W \nonumber \\&\quad + \frac{1}{3} \frac{\partial U_1}{\partial q} \bigg (\frac{q_k+q_{k+1}}{2}\bigg )\Delta W - \frac{2}{3} \frac{\partial U_1}{\partial q} \bigg (\frac{q_k+3 q_{k+1}}{4}\bigg )\Delta W, \end{aligned}$$
  where
  3.35 $$\begin{aligned} P_1&= p_k - \frac{1}{2} \frac{\partial U_0}{\partial q} \bigg (\frac{3 q_k+q_{k+1}}{4}\bigg )\Delta t + \frac{1}{6} \frac{\partial U_0}{\partial q} \bigg (\frac{q_k+q_{k+1}}{2}\bigg )\Delta t \nonumber \\&\quad - \frac{1}{6} \frac{\partial U_0}{\partial q} \bigg (\frac{q_k+3 q_{k+1}}{4}\bigg )\Delta t - \frac{1}{2} \frac{\partial U_1}{\partial q} \bigg (\frac{3 q_k+q_{k+1}}{4}\bigg )\Delta W \nonumber \\&\quad + \frac{1}{6} \frac{\partial U_1}{\partial q} \bigg (\frac{q_k+q_{k+1}}{2}\bigg )\Delta W - \frac{1}{6} \frac{\partial U_1}{\partial q} \bigg (\frac{q_k+3 q_{k+1}}{4}\bigg )\Delta W, \end{aligned}$$
  and $H(q,p)=T_0(p)+U_0(q)$ and $h(q,p)=T_1(p)+U_1(q)$. In this case only the first equation needs to be solved for $q_{k+1}$, and then the second equation is an explicit update.

#### Hamiltonian function *h*(*q*) independent of momentum

In case the Hamiltonian function $h=h(q)$ is independent of the momentum variable *p*, the term $\partial h / \partial p$ does not enter Eq. (3.8d), and therefore we can allow a choice of quadrature rules such that $\alpha _i=0$ or $\beta _i=0$ for some *i*. If $\alpha _i=0$, however, the system (3.8) becomes underdetermined, but at the same time the corresponding $P_i$ does not enter any of the remaining equations, therefore we can simply ignore it. To simplify the notation, let $i_1<\cdots <i_{{\bar{r}}}$ be the set of indices such that $\alpha _{i_m}\not = 0$, and denote ${\bar{\alpha }}_m\equiv \alpha _{i_m}$, ${\bar{c}}_m\equiv c_{i_m}$ for $m=1,\ldots ,{\bar{r}}$. Similarly, let $j_1<\cdots <j_{\tilde{r}}$ be the set of indices such that $\beta _{j_m}\not = 0$, and denote $\tilde{\beta }_m\equiv \beta _{i_m}$, $\tilde{c}_m\equiv c_{j_m}$ for $m=1,\ldots ,\tilde{r}$. In (3.8) leave out the terms and equations corresponding to $\alpha _i=0$ or $\beta _i=0$, and replace $\alpha _i$, $\beta _i$, $c_i$ and *r* by ${\bar{\alpha }}_i$, $\tilde{\beta }_i$, ${\bar{c}}_i$, $\tilde{c}_i$, ${\bar{r}}$ and $\tilde{r}$, accordingly. In other words, this is equivalent to using the quadrature rules $({\bar{\alpha }}_i,{\bar{c}}_i)_{i=1}^{{\bar{r}}}$ and $(\tilde{\beta }_i,\tilde{c}_i)_{i=1}^{\tilde{r}}$ in (3.6). We then simultaneously solve (3.8a), (3.8b) and (3.8d) [$(s+{\bar{r}})N$ equations] for $q^1, \ldots , q^s$ and $P_1,\ldots , P_{{\bar{r}}}$ [$(s+{\bar{r}})N$ unknowns]. A few examples of such integrators are listed below.

1. *P*1*N*1*Q*1*Rec* (*Stochastic symplectic Euler method*)
  The rectangle rule (${\bar{r}}=1$, ${\bar{c}}_1=1$, ${\bar{\alpha }}_1=1$) does not yield a convergent numerical scheme in the general case, but when $h=h(q)$, the Itô and Stratonovich interpretations of (1.1) are equivalent, and the rectangle rule can be used to construct efficient integrators. In fact, applying the rectangle rule to both the Lebesgue and Stratonovich integrals and using polynomials of degree $s=1$ yield a method which can be written as
  3.36 $$\begin{aligned} q_{k+1}&= q_k + \frac{\partial H}{\partial p} \big (q_{k+1}, p_k \big )\Delta t, \nonumber \\ p_{k+1}&= p_k - \frac{\partial H}{\partial q} \big (q_{k+1}, p_k \big )\Delta t - \frac{\partial h}{\partial q} \big (q_{k+1}\big )\Delta W. \end{aligned}$$
  This method is a straightforward generalization of the symplectic Euler scheme (see [18, 40]) and is particularly computationally efficient, as only the first equation needs to be solved for $q_{k+1}$, and then the second equation is an explicit update. Moreover, in case the Hamiltonian *H* is separable, the method becomes explicit.
2. *P*1*N*1*Q*1*RecN*2*Q*2*Lob*
  The accuracy of the stochastic symplectic Euler scheme above can be improved by applying the trapezoidal rule to the Stratonovich integral instead of the rectangle rule. The resulting integrator takes the form
  3.37 $$\begin{aligned} q_{k+1}&= q_k + \frac{\partial H}{\partial p} \big (q_{k+1}, P_1 \big )\Delta t, \nonumber \\ p_{k+1}&= p_k - \frac{\partial H}{\partial q} \big (q_{k+1},P_1 \big )\Delta t - \frac{1}{2} \frac{\partial h}{\partial q} \big (q_k\big )\Delta W - \frac{1}{2} \frac{\partial h}{\partial q} \big (q_{k+1} \big )\Delta W, \end{aligned}$$
  where
  3.38 $$\begin{aligned} P_1= p_k - \frac{1}{2} \frac{\partial h}{\partial q} \big (q_k \big ) \Delta W. \end{aligned}$$
  While having a similar computational cost, this method yields a more accurate solution than (3.36) (see Sect. 4 for numerical tests). Moreover, in case the Hamiltonian *H* is separable, the method becomes explicit.
3. *P*1*N*1*Q*1*RecN*1*Q*2*Gau*
  Similarly, if we apply the midpoint rule instead of the trapezoidal rule, we obtain the following modification of the stochastic symplectic Euler method:
  3.39 $$\begin{aligned} q_{k+1}&= q_k + \frac{\partial H}{\partial p} \big (q_{k+1}, P_1 \big )\Delta t, \nonumber \\ p_{k+1}&= p_k - \frac{\partial H}{\partial q} \big (q_{k+1},P_1 \big )\Delta t - \frac{\partial h}{\partial q} \bigg ( \frac{q_k+q_{k+1}}{2}\bigg )\Delta W, \end{aligned}$$
  where
  3.40 $$\begin{aligned} P_1= p_k - \frac{1}{2} \frac{\partial h}{\partial q} \bigg ( \frac{q_k+q_{k+1}}{2}\bigg ) \Delta W. \end{aligned}$$
  This method demonstrates a similar performance as (3.37) (see Sect. 4 for numerical tests). It becomes explicit if the Hamiltonian *H* is separable and the noise is additive, i.e., $\partial h / \partial q = \text {const}$.
4. *P*2*N*2*Q*2*LobN*1*Q*1*Rec*
  A modification of the stochastic Störmer–Verlet method (3.29) is obtained if we use the rectangle rule to approximate the Stratonovich integral:
  3.41 $$\begin{aligned} P_1&= p_k - \frac{1}{2} \frac{\partial H}{\partial q} \big (q_k, P_1 \big )\Delta t, \nonumber \\ q_{k+1}&= q_k + \frac{1}{2} \frac{\partial H}{\partial p} \big (q_k, P_1 \big )\Delta t + \frac{1}{2} \frac{\partial H}{\partial p} \big (q_{k+1}, P_1 \big )\Delta t, \nonumber \\ p_{k+1}&= P_1 - \frac{1}{2} \frac{\partial H}{\partial q} \big (q_{k+1}, P_1 \big )\Delta t - \frac{\partial h}{\partial q} \big (q_{k+1}\big )\Delta W. \end{aligned}$$
  This integrator has a similar computational cost as the stochastic Störmer–Verlet method (see Sect. 4), but it yields a slightly less accurate solution (see Sect. 4). Moreover, in case the Hamiltonian *H* is separable, the method becomes explicit.
5. *P*1*N*1*Q*2*GauN*2*Q*2*Lob*
  This integrator is a modification of the stochastic midpoint method (3.28). We apply the midpoint rule (${\bar{r}}=1$, ${\bar{c}}_1=1/2$, ${\bar{\alpha }}_1=1$) to the Lebesgue integral in (3.4), and the trapezoidal rule ($\tilde{r}=2$, $\tilde{c}_1=0$, $\tilde{c}_2=1$, $\tilde{\beta }_1=1/2$, $\tilde{\beta }_2=1/2$) to the Stratonovich integral. The resulting numerical scheme can be written as
  3.42 $$\begin{aligned} q_{k+1}&= q_k + \frac{\partial H}{\partial p} \bigg (\frac{q_k+q_{k+1}}{2},P_1 \bigg )\Delta t, \nonumber \\ p_{k+1}&= p_k - \frac{\partial H}{\partial q} \bigg (\frac{q_k+q_{k+1}}{2},P_1 \bigg )\Delta t - \frac{1}{2} \frac{\partial h}{\partial q} \big (q_k\big )\Delta W - \frac{1}{2} \frac{\partial h}{\partial q} \big (q_{k+1} \big )\Delta W, \end{aligned}$$
  where
  3.43 $$\begin{aligned} P_1=\frac{p_k+p_{k+1}}{2}+\frac{1}{4} \Delta W \left[ \frac{\partial h}{\partial q} \big (q_{k+1} \big )-\frac{\partial h}{\partial q} \big (q_k \big ) \right] . \end{aligned}$$
  This method is fully implicit, but similar to (3.28), simplifies when the Hamiltonian *H* is separable.
6. *P*1*N*2*Q*2*LobN*1*Q*2*Gau*
  If instead we apply the trapezoidal rule to the Lebesgue integral and the midpoint rule to the Stratonovich integral in (3.4), we obtain a modification of the stochastic trapezoidal rule (3.30):
  3.44 $$\begin{aligned} p_k&= \frac{1}{2}(P_1+P_2) + \frac{1}{2} \frac{\partial H}{\partial q} \big (q_k, P_1 \big )\Delta t + \frac{1}{2} \frac{\partial h}{\partial q} \bigg (\frac{q_k+q_{k+1}}{2} \bigg )\Delta W, \nonumber \\ p_{k+1}&= \frac{1}{2}(P_1+P_2) - \frac{1}{2} \frac{\partial H}{\partial q} \big (q_{k+1}, P_2 \big )\Delta t - \frac{1}{2} \frac{\partial h}{\partial q} \bigg (\frac{q_k+q_{k+1}}{2} \bigg )\Delta W, \nonumber \\ q_{k+1}&= q_k + \frac{\partial H}{\partial p} \big (q_k,P_1 \big )\Delta t, \nonumber \\ q_{k+1}&= q_k + \frac{\partial H}{\partial p} \big (q_{k+1},P_2 \big )\Delta t. \end{aligned}$$
  This method becomes explicit when the Hamiltonian *H* is separable and the noise is additive, i.e., $\partial h / \partial q = \text {const}$.

### Convergence

Various criteria for convergence of stochastic schemes have been suggested in the literature (see [28, 42]). Some criteria concentrate on pathwise approximations of the exact solutions (*mean-square convergence, strong convergence*), while others focus on approximations of some functionals instead (*weak convergence*). We are here primarily interested in mean-square convergence. Let ${\bar{z}}(t) = ({\bar{q}}(t),{\bar{p}}(t))$ be the exact solution to (1.1) with the initial conditions $q_0$ and $p_0$, and let $z_k = (q_k,p_k)$ denote the numerical solution at time $t_k$ obtained by applying (3.8) iteratively *k* times with the constant time step $\Delta t$. The numerical solution is said to converge in the mean-square sense with global order *r* if there exist $\delta >0$ and a constant $C>0$ such that for all $\Delta t \in (0,\delta )$ we have

3.45 $$\begin{aligned} \sqrt{E(\Vert z_K-{\bar{z}}(T)\Vert ^2 )} \le C\Delta t^r, \end{aligned}$$

where $T = K\Delta t$, as defined before, and *E* denotes the expected value. In principle, in order to determine the mean-square order of convergence of the Galerkin variational integrator (3.8) we need to calculate the power series expansions of $q_{k+1}$ and $p_{k+1}$ in terms of the powers of $\Delta t$ and $\Delta W$, and compare them to the Stratonovich–Taylor expansions for the exact solution ${\bar{q}}(t_k+\Delta t)$ and ${\bar{p}}(t_k+\Delta t)$ (see [12, 28, 42]). It is quite a tedious task to do in the general case, therefore we only discuss the examples presented in Sect. 3.4. For instance, in case of the stochastic trapezoidal method (3.30) we plug in series expansions for $P_1$, $P_2$, $q_{k+1}$ and $p_{k+1}$, and determine their coefficients by expanding the derivatives of the Hamiltonians into Taylor series around $(q_k,p_k)$ and comparing the terms corresponding to the like powers of $\Delta t$ and $\Delta W$. We find that

3.46 $$\begin{aligned} q_{k+1}&= q_k + \frac{\partial H}{\partial p}\Delta t + \frac{\partial h}{\partial p}\Delta W + \frac{1}{2} \bigg (\frac{\partial ^2 h}{\partial p \partial q} \frac{\partial h}{\partial p} - \frac{\partial ^2 h}{\partial p^2} \frac{\partial h}{\partial q} \bigg ) \Delta W^2+\cdots , \nonumber \\ p_{k+1}&= p_k - \frac{\partial H}{\partial q}\Delta t - \frac{\partial h}{\partial q}\Delta W - \frac{1}{2} \bigg (\frac{\partial ^2 h}{\partial q^2} \frac{\partial h}{\partial p} - \frac{\partial ^2 h}{\partial q \partial p} \frac{\partial h}{\partial q}\bigg ) \Delta W^2+\cdots , \end{aligned}$$

where the derivatives of the Hamiltonians are evaluated at $(q_k,p_k)$. Let ${\bar{q}}(t;q_k,p_k)$ and ${\bar{p}}(t;q_k,p_k)$ denote the exact solution of (1.1) such that ${\bar{q}}(t_k;q_k,p_k)=q_k$ and ${\bar{p}}(t_k;q_k,p_k)=p_k$. Using (1.1) we calculate the Stratonovich–Taylor expansions for ${\bar{q}}(t_{k+1};q_k,p_k)$ and ${\bar{p}}(t_{k+1};q_k,p_k)$, and comparing them to (3.46) we find that

3.47 $$\begin{aligned} E\big (q_{k+1}-{\bar{q}}(t_{k+1};q_k,p_k)\big )&= O(\Delta t^2), \qquad \sqrt{E\big ( \Vert q_{k+1}-{\bar{q}}(t_{k+1};q_k,p_k)\Vert ^2 \big )} = O(\Delta t^{\frac{3}{2}}), \nonumber \\ E\big (p_{k+1}-{\bar{p}}(t_{k+1};q_k,p_k)\big )&= O(\Delta t^2), \qquad \sqrt{E\big ( \Vert p_{k+1}-{\bar{p}}(t_{k+1};q_k,p_k)\Vert ^2 \big )} = O(\Delta t^{\frac{3}{2}}). \end{aligned}$$

Using Theorem 1.1 from [42], we conclude that the stochastic trapezoidal method (3.30) has mean-square order of convergence $r=1$. In a similar fashion we prove that all methods presented in Sect. 3.4 are convergent with mean-square order 1. We further verify these results numerically in Sect. 4.1.

#### Remark

For simplicity and clarity of the exposition, in this work we are mainly concerned with a one-dimensional noise in (1.1). However, all of the constructions and results presented in Sects. 2 and 3 generalize in a straightforward manner, when a multidimensional noise $W^1, W^2, \ldots , W^M$, together with the corresponding Hamiltonian functions $h_1, h_2, \ldots , h_M$, is considered in (1.1), except that the integrators derived in Sect. 3.4 in general do not attain mean-square order 1.0 of convergence, unless the noise is commutative. Indeed, if we repeat the procedure described above for the stochastic trapezoidal method, we will obtain the following power series expansions in terms of the powers of $\Delta t$ and $\Delta W^i$:

3.48 $$\begin{aligned} q_{k+1}&= q_k + \frac{\partial H}{\partial p}\Delta t + \sum _{i=1}^M \frac{\partial h_i}{\partial p}\Delta W^i + \frac{1}{2} \sum _{i=1}^M \varGamma _{ii} (\Delta W^i)^2 \nonumber \\&\quad + \frac{1}{2}\sum _{i=1}^M \sum _{\begin{array}{c} j=1 \\ j \not = i \end{array}}^M \varGamma _{ij} \Delta W^i \Delta W^j + \cdots , \nonumber \\ p_{k+1}&= p_k - \frac{\partial H}{\partial q}\Delta t - \sum _{i=1}^M \frac{\partial h_i}{\partial q}\Delta W^i + \frac{1}{2} \sum _{i=1}^M \varLambda _{ii} (\Delta W^i)^2 \nonumber \\&\quad + \frac{1}{2}\sum _{i=1}^M \sum _{\begin{array}{c} j=1 \\ j \not = i \end{array}}^M \varLambda _{ij} \Delta W^i \Delta W^j +\cdots , \end{aligned}$$

where the vectors $\varGamma _{ij}$ and $\varLambda _{ij}$ for each $i,j=1,\ldots ,M$ are defined as

3.49 $$\begin{aligned} \varGamma _{ij} = \frac{\partial ^2 h_j}{\partial p \partial q} \frac{\partial h_i}{\partial p} - \frac{\partial ^2 h_j}{\partial p^2} \frac{\partial h_i}{\partial q}, \qquad \varLambda _{ij} = -\frac{\partial ^2 h_j}{\partial q^2} \frac{\partial h_i}{\partial p} + \frac{\partial ^2 h_j}{\partial q \partial p} \frac{\partial h_i}{\partial q}, \end{aligned}$$

and the derivatives of the Hamiltonians are evaluated at $(q_k, p_k)$. On the other hand, the Stratonovich–Taylor expansions for ${\bar{q}}(t_{k+1};q_k,p_k)$ and ${\bar{p}}(t_{k+1};q_k,p_k)$ read, respectively,

3.50 $$\begin{aligned} {\bar{q}}(t_{k+1};q_k,p_k)&= q_k + \frac{\partial H}{\partial p}\Delta t + \sum _{i=1}^M \frac{\partial h_i}{\partial p}\Delta W^i + \frac{1}{2} \sum _{i=1}^M \varGamma _{ii} (\Delta W^i)^2 \nonumber \\&\quad + \sum _{i=1}^M \sum _{\begin{array}{c} j=1 \\ j \not = i \end{array}}^M \varGamma _{ij} J_{ij} + \cdots , \nonumber \\ {\bar{p}}(t_{k+1};q_k,p_k)&= p_k - \frac{\partial H}{\partial q}\Delta t - \sum _{i=1}^M \frac{\partial h_i}{\partial q}\Delta W^i + \frac{1}{2} \sum _{i=1}^M \varLambda _{ii} (\Delta W^i)^2 \nonumber \\&\quad + \sum _{i=1}^M \sum _{\begin{array}{c} j=1 \\ j \not = i \end{array}}^M \varLambda _{ij} J_{ij} +\cdots , \end{aligned}$$

where $J_{ij} = \int _{t_k}^{t_{k+1}}\int _{t_k}^{t}dW^i(\tau )\circ dW^j(t)$ denotes a double Stratonovich integral. Comparing (3.49) and (3.50), we find that in the general case not all first order terms agree, and therefore we only have the local error estimates

3.51 $$\begin{aligned} E\big (q_{k+1}-{\bar{q}}(t_{k+1};q_k,p_k)\big )&= O(\Delta t^{\frac{3}{2}}), \qquad \sqrt{E\big ( \Vert q_{k+1}-{\bar{q}}(t_{k+1};q_k,p_k)\Vert ^2 \big )} = O(\Delta t), \nonumber \\ E\big (p_{k+1}-{\bar{p}}(t_{k+1};q_k,p_k)\big )&= O(\Delta t^{\frac{3}{2}}), \qquad \sqrt{E\big ( \Vert p_{k+1}-{\bar{p}}(t_{k+1};q_k,p_k)\Vert ^2 \big )} = O(\Delta t). \end{aligned}$$

Theorem 1.1 from [42] then implies that the stochastic trapezoidal method has mean-square order 1 / 2. However, if the noise is commutative, that is, if the following conditions are satisfied

3.52 $$\begin{aligned} \varGamma _{ij}=\varGamma _{ji}, \qquad \varLambda _{ij}=\varLambda _{ji}, \quad \text {for all }i,j=1,\ldots ,M, \end{aligned}$$

then using the property $J_{ij}+J_{ji}=\Delta W^i \Delta W^j$ (see [28, 42]), one can easily show

3.53 $$\begin{aligned} \sum _{i=1}^M \sum _{\begin{array}{c} j=1 \\ j \not = i \end{array}}^M \varGamma _{ij} J_{ij} = \frac{1}{2}\sum _{i=1}^M \sum _{\begin{array}{c} j=1 \\ j \not = i \end{array}}^M \varGamma _{ij} \Delta W^i \Delta W^j, \qquad \quad \sum _{i=1}^M \sum _{\begin{array}{c} j=1 \\ j \not = i \end{array}}^M \varLambda _{ij} J_{ij} = \frac{1}{2}\sum _{i=1}^M \sum _{\begin{array}{c} j=1 \\ j \not = i \end{array}}^M \varLambda _{ij} \Delta W^i \Delta W^j. \end{aligned}$$

In that case all first-order terms in the expansions (3.48) and (3.50) agree, and we again have the local error estimates (3.47), meaning that the scheme has mean-square order 1.0. Similar analysis holds for all the methods presented in Sect. 3.4. It should be noted that the commutation conditions (3.52) hold for two important special cases:

- Hamiltonian functions $h_i$ linear in *q* and *p* for all $i=1,\ldots ,M$, i.e. additive noise
- Hamiltonian functions $h_i$ simultaneously independent of one of the variables *q* or *p* for all $i=1,\ldots ,M$

The latter in particular means that the methods presented in Sect. 3.4.2 retain their mean-square order of convergence for multidimensional noises.

### Methods of order 3 / 2

In order to construct stochastic Galerkin variational integrators of higher order one needs to include higher order terms in the discretization of the Stratonovich integral in (3.5). For example, a method of mean-square order 3/2 must include terms involving $\Delta Z = \int _{t_k}^{t_{k+1}}\int _{t_k}^{t}dW(\xi )\,dt$ (see [12, 43, 44]). Inspired by the theory presented in [12], we can add extra terms to the discrete Hamiltonian (3.6) and write it as

3.54 $$\begin{aligned}&H^+_d(q_k,p_{k+1}) \nonumber \\&\quad =\mathop {\hbox {ext}}_{ \begin{array}{c} q^1,\ldots ,q^s \in Q \\ P_1, \ldots , P_r \in Q^* \\ q^0 = q_k \end{array} } \left\{ p_{k+1}q^s - \Delta t \sum _{i=1}^r \alpha _i \Big [ P_i {\dot{q}}_d(t_k+c_i\Delta t) - H\big (q_d(t_k+c_i\Delta t),P_i\big ) \Big ]\right. \nonumber \\&\qquad \left. + \Delta W \sum _{i=1}^r \beta _i h\big (q_d(t_k+c_i\Delta t),P_i\big ) + \frac{\Delta Z}{\Delta t} \sum _{i=1}^r \gamma _i h\big (q_d(t_k+c_i\Delta t),P_i\big ) \right\} . \end{aligned}$$

The random variables $\Delta W$ and $\Delta Z$ have a Gaussian joint distribution (see [28, 44]), and at each time step they can be simulated by two independent $\mathscr {N}(0,1)$-distributed random variables $\chi$ and $\eta$ as

3.55 $$\begin{aligned} \Delta W = \chi \sqrt{\Delta t}, \qquad \Delta Z = \frac{1}{2}\Delta t^{\frac{3}{2}} \left( \chi + \frac{1}{\sqrt{3}} \eta \right) . \end{aligned}$$

In order to achieve mean-square convergence of order 3/2 one needs to determine appropriate values for the parameters *s*, *r*, $\alpha _i$, $\beta _i$, $\gamma _i$, and $c_i$. However, we will not attempt to achieve this in the present work. Instead, we will show that some known stochastic symplectic integrators can be derived as stochastic Galerkin variational integrators.

Suppose the Hamiltonian is separable, i.e., $H(q,p)=T(p)+U(q)$, and the Hamiltonian function $h=h(q)$ does not depend on momentum. Consider the discrete Hamiltonian

3.56 $$\begin{aligned}&H^+_d(q_k,p_{k+1})= \nonumber \\&\quad =\mathop {\hbox {ext}}_{ \begin{array}{c} q^1,\ldots ,q^s \in Q \\ P_1, \ldots , P_r \in Q^* \\ q^0 = q_k \end{array} } \left\{ p_{k+1}q^s - \Delta t \sum _{i=1}^r \Big [ {\bar{\alpha }}_i P_i {\dot{q}}_d(t_k+c_i\Delta t) - {\bar{\alpha }}_i U\big (q_d(t_k+c_i\Delta t)\big ) - \alpha _i T\big (P_i\big ) \Big ]\right. \nonumber \\&\qquad \left. + \Delta W \sum _{i=1}^r {\bar{\beta }}_i h\big (q_d(t_k+c_i\Delta t)\big ) + \frac{\Delta Z}{\Delta t} \sum _{i=1}^r {\bar{\gamma }}_i h\big (q_d(t_k+c_i\Delta t)\big ) \right\} , \end{aligned}$$

where different weights ${\bar{\alpha }}_i$ and $\alpha _i$ were applied to the potential *U*(*q*) and kinetic *T*(*p*) terms, respectively. Similar to (3.8), the corresponding stochastic variational integrator takes the form

3.57 $$\begin{aligned} -p_k&=\sum _{i=1}^r {\bar{\alpha }}_i \Big [ P_i {\dot{l}}_{0,s}(c_i) - \Delta t \frac{\partial U}{\partial q}\big (t_k+c_i\Delta t\big ) l_{0,s}(c_i) \Big ] \nonumber \\&\quad - \sum _{i=1}^r \Big ({\bar{\beta }}_i \Delta W + {\bar{\gamma }}_i\frac{\Delta Z}{\Delta t}\Big ) \frac{\partial h}{\partial q}\big (t_k+c_i\Delta t\big ) l_{0,s}(c_i), \nonumber \\ 0&=\sum _{i=1}^r {\bar{\alpha }}_i \Big [ P_i {\dot{l}}_{\mu ,s}(c_i) - \Delta t \frac{\partial U}{\partial q}\big (t_k+c_i\Delta t\big ) l_{\mu ,s}(c_i) \Big ] \nonumber \\&\quad - \sum _{i=1}^r \Big ({\bar{\beta }}_i \Delta W + {\bar{\gamma }}_i\frac{\Delta Z}{\Delta t}\Big ) \frac{\partial h}{\partial q}\big (t_k+c_i\Delta t\big ) l_{\mu ,s}(c_i), \nonumber \\ p_{k+1}&=\sum _{i=1}^r {\bar{\alpha }}_i \Big [ P_i {\dot{l}}_{s,s}(c_i) - \Delta t \frac{\partial U}{\partial q}\big (t_k+c_i\Delta t\big ) l_{s,s}(c_i) \Big ] \nonumber \\&\quad - \sum _{i=1}^r \Big ({\bar{\beta }}_i \Delta W + {\bar{\gamma }}_i\frac{\Delta Z}{\Delta t}\Big ) \frac{\partial h}{\partial q}\big (t_k+c_i\Delta t\big ) l_{s,s}(c_i), \nonumber \\ {\bar{\alpha }}_i {\dot{q}}_d&(t_k+c_i\Delta t) = \alpha _i \frac{\partial T}{\partial p}\big (P_i\big ), \nonumber \\ q_{k+1}&=q^s, \end{aligned}$$

where $\mu =1,\ldots ,s-1$ in the second equation, and $i=1,\ldots , r$ in the fourth equation. In the special case when $r=s$ and

3.58 $$\begin{aligned} {\bar{\alpha }}_i = \int _0^1 {\bar{l}}_{i,s-1}(\tau )\,d\tau , \quad i=1,\ldots ,s, \end{aligned}$$

we can show, similar to Theorem 3.4, that the stochastic Galerkin variational integrator (3.57) is equivalent to the stochastic partitioned Runge–Kutta method

3.59 $$\begin{aligned} Q_i&= q_k + \Delta t \sum _{j=1}^s a_{ij} \frac{\partial T}{\partial p}(P_j), \quad i=1,\ldots ,s, \nonumber \\ P_i&\!=\! p_k \!-\! \Delta t \sum _{j=1}^s {\bar{a}}_{ij} \frac{\partial U}{\partial q}(Q_j) - \sum _{j=1}^s \left( {\bar{b}}_{ij} \Delta W + {\bar{\lambda }}_{ij}\frac{\Delta Z}{\Delta t} \right) \frac{\partial h}{\partial q}(Q_j), \quad i\!=\!1,\ldots ,s, \nonumber \\ q_{k+1}&= q_k + \Delta t \sum _{i=1}^s \alpha _i \frac{\partial T}{\partial p}(P_i),\nonumber \\ p_{k+1}&= p_k - \Delta t \sum _{i=1}^s {\bar{\alpha }}_i \frac{\partial U}{\partial q}(Q_i) - \sum _{i=1}^s \left( {\bar{\beta }}_i \Delta W + {\bar{\gamma }}_i \frac{\Delta Z}{\Delta t} \right) \frac{\partial h}{\partial q}(Q_i), \end{aligned}$$

with the coefficients

3.60 $$\begin{aligned} a_{ij}&=\frac{\alpha _j}{{\bar{\alpha }}_j}\int _0^{c_i} {\bar{l}}_{j,s-1}(\tau )\,d\tau ,&{\bar{a}}_{ij}&= \frac{{\bar{\alpha }}_j(\alpha _i-a_{ji})}{\alpha _i}, \nonumber \\ {\bar{b}}_{ij}&= \frac{{\bar{\beta }}_j(\alpha _i-a_{ji})}{\alpha _i},&{\bar{\lambda }}_{ij}&= \frac{{\bar{\gamma }}_j(\alpha _i-a_{ji})}{\alpha _i}, \quad i,j=1,\ldots ,s, \end{aligned}$$

where we assume $\alpha _i \not = 0$ and ${\bar{\alpha }}_i \not = 0$ for all *i*. Partitioned Runge–Kutta methods of type (3.59) were considered in [44]. In particular, it was shown that for $s=2$ the choice of the coefficients

3.61 $$\begin{aligned} \alpha _1&=2/3,&\alpha _2&=1/3,&{\bar{\alpha }}_1&=1/4,&{\bar{\alpha }}_2&=3/4, \nonumber \\ a_{11}&= 0,&a_{12}&=0,&{\bar{a}}_{11}&=1/4,&{\bar{a}}_{12}&=0, \nonumber \\ a_{21}&= 2/3,&a_{22}&=0,&{\bar{a}}_{21}&=1/4,&{\bar{a}}_{22}&=3/4, \nonumber \\ {\bar{\beta }}_1&= -1/2,&{\bar{\beta }}_2&=3/2,&{\bar{\gamma }}_1&=3/2&{\bar{\gamma }}_2&=-3/2, \nonumber \\ {\bar{b}}_{11}&=-1/2,&{\bar{b}}_{12}&=0,&{\bar{\lambda }}_{11}&=3/2,&{\bar{\lambda }}_{12}&=0, \nonumber \\ {\bar{b}}_{21}&=-1/2,&{\bar{b}}_{22}&=3/2,&{\bar{\lambda }}_{21}&=3/2,&{\bar{\lambda }}_{22}&=-3/2, \end{aligned}$$

gives a method of mean-square order 3/2 (see Theorem 4.3 in [44]).

## Numerical experiments

In this section we present the results of our numerical experiments. We verify numerically the convergence results from Sect. 3.5 and investigate the conservation properties of our integrators. In particular, we show that our stochastic variational integrators demonstrate superior behavior in long-time simulations compared to some popular general purpose non-symplectic stochastic algorithms.

### Numerical convergence analysis

#### Kubo oscillator

In order to test the convergence of the numerical algorithms from Sect. 3.4.1 we performed computations for the Kubo oscillator, which is defined by $H(q,p)=p^2/2+q^2/2$ and $h(q,p)=\beta (p^2/2+q^2/2)$, where $\beta$ is the noise intensity (see [44]). The Kubo oscillator is used in the theory of magnetic resonance and laser physics. The exact solution is given by

**Fig. 1:**
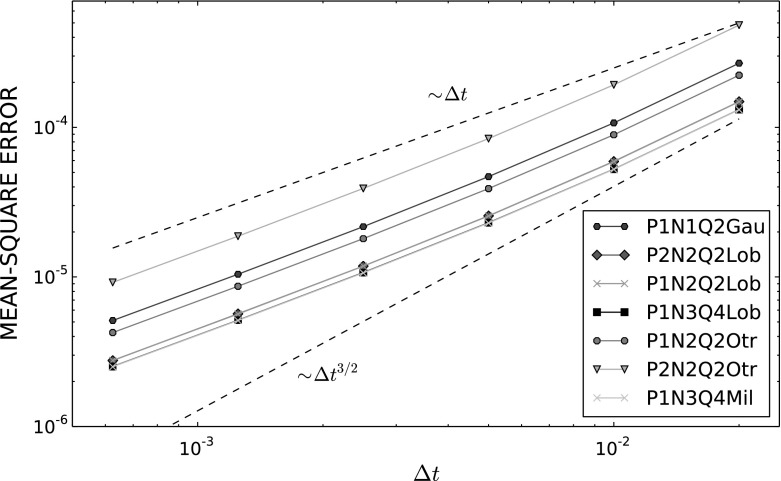
The mean-square error at the time $T=3.2$ as a function of the time step $\Delta t$ for the simulations of the Kubo oscillator with the initial conditions $q_0=0$, $p_0=1$ and the noise intensity $\beta =0.1$. In each case 2000 sample paths were generated. The tested integrators proved to be convergent of order 1.0 in the mean-square sense. Note that the plots for *P*2*N*2*Q*2*Lob* and *P*1*N*2*Q*2*Lob*, as well as for *P*1*N*3*Q*4*Lob* and *P*1*N*3*Q*4*Mil*, overlap very closely

4.1 $$\begin{aligned} {\bar{q}}(t)&=p_0 \sin (t+\beta W(t)) + q_0 \cos (t+\beta W(t)), \nonumber \\ {\bar{p}}(t)&=p_0 \cos (t+\beta W(t)) - q_0 \sin (t+\beta W(t)), \end{aligned}$$

where $q_0$ and $p_0$ are the initial conditions. Simulations with the initial conditions $q_0=0$, $p_0=1$ and the noise intensity $\beta =0.1$ were carried out until the time $T=3.2$ for a number of time steps $\Delta t = 0.000625, 0.00125, 0.0025, 0.005, 0.01, 0.02$. In each case 2000 sample paths were generated. Let $z_{\Delta t}(t) = (q_{\Delta t}(t), p_{\Delta t}(t) )$ denote the numerical solution. We used the exact solution (4.1) as a reference for computing the mean-square error $\sqrt{E(|z_{\Delta t}(T)-{\bar{z}}(T)|^2)}$, where ${\bar{z}}(t) = ({\bar{q}}(t), {\bar{p}}(t) )$. The dependence of this error on the time step $\Delta t$ is depicted in Fig. 1. We verified that our algorithms have mean-square order of convergence 1.0. The integrators *P*1*N*3*Q*4*Lob*, *P*1*N*3*Q*4*Mil*, *P*1*N*2*Q*2*Lob* (stochastic trapezoidal method), and *P*2*N*2*Q*2*Lob* (stochastic Störmer–Verlet method) turned out to be the most accurate, with the latter two having least computational cost.

#### Synchrotron oscillations of particles in storage rings

**Fig. 2:**
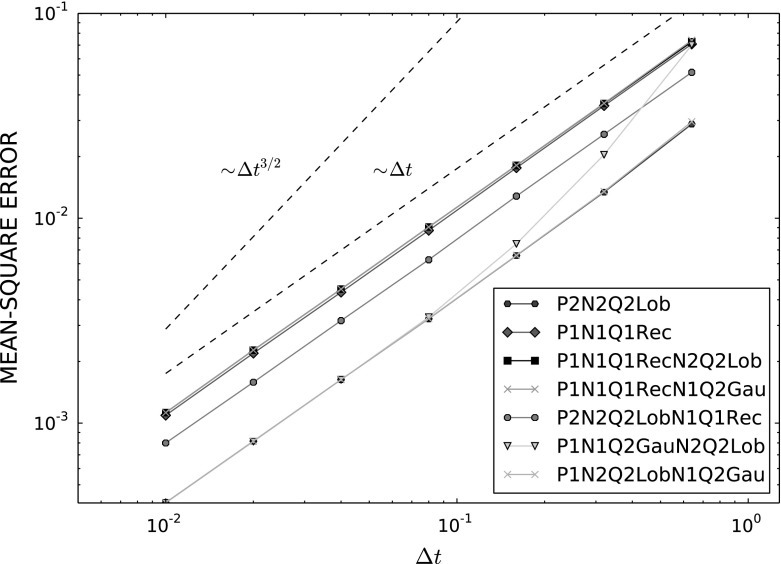
The mean-square error at the time $T=3.2$ as a function of the time step $\Delta t$ for the simulations of the synchrotron oscillations of a particle in a storage ring with the initial conditions $q_0=0$, $p_0=1$ and the noise intensity $\beta =0.1$. In each case 2000 sample paths were generated. The tested integrators proved to be convergent of order 1.0 in the mean-square sense. Note that the plots for *P*1*N*1*Q*1*Rec*, *P*1*N*1*Q*1*RecN*2*Q*2*Lob*, and *P*1*N*1*Q*1*RecN*1*Q*2*Gau*, as well as for *P*2*N*2*Q*2*Lob* and *P*1*N*2*Q*2*LobN*1*Q*2*Gau*, overlap very closely

We carried out a similar test for the numerical schemes from Sect. 3.4.2. We performed computations for the stochastic Hamiltonian system defined by $H(q,p)=p^2/2-\cos q$ and $h(q)=\beta \sin q$, where $\beta$ is the noise intensity. Systems of this type are used for modeling synchrotron oscillations of a particle in a storage ring. Due to fluctuating electromagnetic fields, a particle performs stochastically perturbed oscillations with respect to a reference particle which travels with fixed energy along the design orbit of the accelerator; in this description *p* corresponds to the energy deviation of the particle from the reference particle, and *q* measures the longitudinal phase difference of both particles (see [17, 56] for more details). Simulations with the initial conditions $q_0=0$, $p_0=1$ and the noise intensity $\beta =0.1$ were carried out until the time $T=3.2$ for a number of time steps $\Delta t = 0.01, 0.02, 0.04, 0.08, 0.16, 0.32, 0.64$. In each case 2000 sample paths were generated. The mean-square error was calculated with respect to a high-precision reference solution generated using the order 3/2 strong Taylor scheme (see [28], Chapter 10.4) with a very fine time step $\Delta t = 2\cdot 10^{-6}$. The dependence of this error on the time step $\Delta t$ is depicted in Fig. 2. We verified that our algorithms have mean-square order of convergence 1.0.

**Fig. 3:**
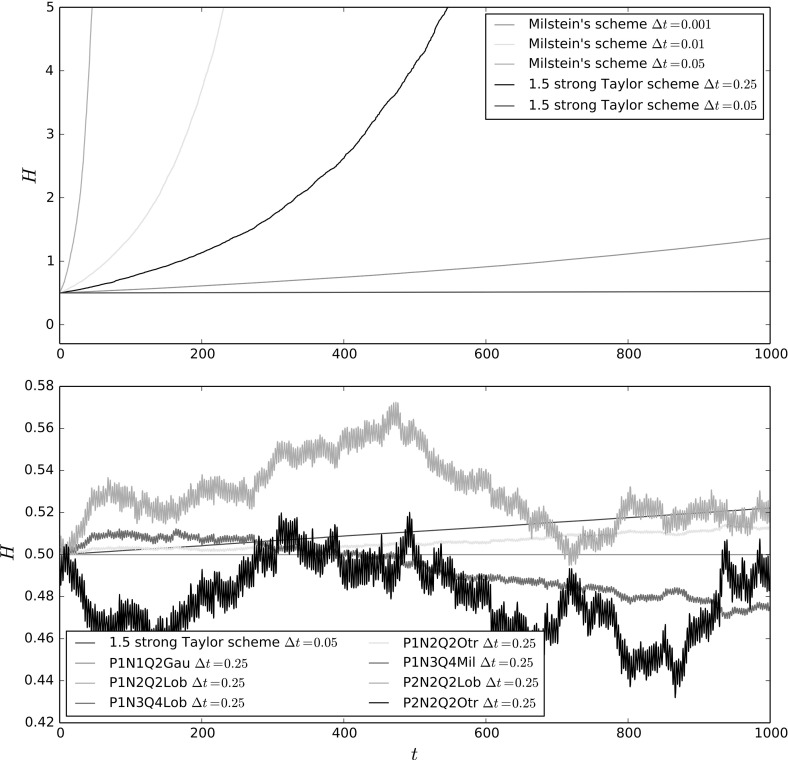
The numerical Hamiltonian for the simulations of the Kubo oscillator with the initial conditions $q_0=0$, $p_0=1$ and the noise intensity $\beta =0.1$. Top The results obtained with Milstein’s scheme and the order 3/2 strong Taylor scheme. We see that the Hamiltonian tends to blow up despite using small time steps. Bottom The results obtained with the integrators derived in Sect. 3.4.1. For comparison, the solution obtained with the Taylor scheme for $\Delta t=0.05$ is also included. Note that for clarity the same color code is applied when the plots for some integrators overlap very closely (color figure online)

### Long-time energy behavior

#### Kubo oscillator

One can easily check that in the case of the Kubo oscillator the Hamiltonian *H*(*q*, *p*) stays constant for almost all sample paths, i.e., $H({\bar{q}}(t), {\bar{p}}(t))=H(q_0,p_0)$ almost surely. We used this example to test the performance of the integrators from Sect. 3.4.1. Simulations with the initial conditions $q_0=0$, $p_0=1$, the noise intensity $\beta =0.1$, and the relatively large time step $\Delta t = 0.25$ were carried out until the time $T=1000$ (approximately 160 periods of the oscillator in the absence of noise) for a single realization of the Wiener process. For comparison, similar simulations were carried out using non-symplectic explicit methods like Milstein’s scheme and the order 3/2 strong Taylor scheme (see [28]). The numerical value of the Hamiltonian *H*(*q*, *p*) as a function of time for each of the integrators is depicted in Fig. 3. We find that the non-symplectic schemes do not preserve the Hamiltonian well, even if small time steps are used. For example, we find that Milstein’s scheme does not give a satisfactory solution even with $\Delta t = 0.001$, and though the Taylor scheme with $\Delta t=0.05$ yields a result comparable to the variational integrators, the growing trend of the numerical Hamiltonian is evident. On the other hand, the variational integrators give numerical solutions for which the Hamiltonian oscillates around the true value (one can check via a direct calculation that the stochastic midpoint method (3.28) in this case preserves the Hamiltonian exactly; of course this does not necessarily hold in the general case).

#### Anharmonic oscillator

In general the Hamiltonian *H*(*q*, *p*) does not stay constant for stochastic Hamilton equations. To determine how well our integrators perform in such cases we considered the anharmonic oscillator defined by $H(q,p)=p^2/2+\gamma q^4$ and $h(q)=\beta q$, where $\beta$ is the noise intensity and $\gamma$ is a parameter. One can calculate the expected value of the Hamiltonian analytically as

**Fig. 4:**
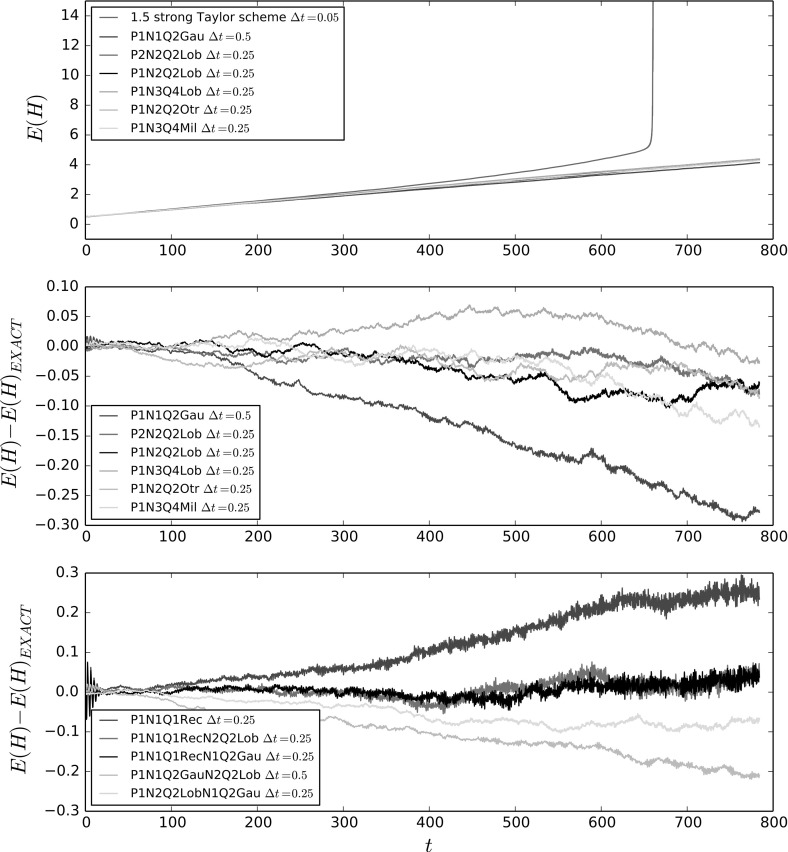
Top The numerical value of the mean Hamiltonian *E*(*H*) for the simulations of the anharmonic oscillator with the initial conditions $q_0=0$, $p_0=1$, the parameter $\gamma =0.1$, and the noise intensity $\beta =0.1$ is shown for the solutions computed with the order 3/2 strong Taylor scheme using the time step $\Delta t=0.05$ and the variational integrators derived in Sect. 3.4.1 using the time step $\Delta t=0.25$ or $\Delta t=0.5$. The variational integrators accurately capture the linear growth of *E*(*H*), whereas the Taylor scheme fails to reproduce that behavior. Middle The difference between the numerical value of the mean Hamiltonian *E*(*H*) and the exact value (4.2) is shown for the integrators derived in Sect. 3.4.1. Bottom Same for the integrators derived in Sect. 3.4.2. The integrators *P*1*N*1*Q*1*RecN*2*Q*2*Lob* and *P*1*N*1*Q*1*RecN*1*Q*2*Gau* prove to be particularly accurate, while having a low computational cost

4.2 $$\begin{aligned} E\Big ( H\big ( q(t),p(t) \big ) \Big ) = H\big ( q_0,p_0 \big ) + \frac{\beta ^2}{2}t, \end{aligned}$$

that is, the mean value of the Hamiltonian grows linearly in time (see [56]). Simulations with the initial conditions $q_0=0$, $p_0=1$, the parameter $\gamma =0.1$, and the noise intensity $\beta =0.1$ were carried out until the time $T=784$ (approximately 100 periods of the oscillator in the absence of noise). In each case 10,000 sample paths were generated. The numerical value of the mean Hamiltonian *E*(*H*) as a function of time for each of the integrators is depicted in Fig. 4. We see that the variational integrators accurately capture the linear growth of *E*(*H*), whereas the Taylor scheme fails to reproduce that behavior even when a smaller time step is used. It is worth noting that the integrators *P*1*N*1*Q*1*RecN*2*Q*2*Lob* and *P*1*N*1*Q*1*RecN*1*Q*2*Gau* yield a very accurate solution, while being computationally efficient, as discussed in Sect. 3.4.2.

##### Remark

One can verify by a direct calculation that when the *P*2*N*2*Q*2*Otr* integrator (example 6 in Sect. 3.4.1) is applied to the Kubo oscillator, then the corresponding system of Eqs. (3.18) does not have a solution when $\Delta t + \beta \Delta W = 3$. To avoid numerical difficulties, one could in principle use the truncated increments (3.9) with, e.g., $A=(3-\Delta t)/(2\beta )$ (for $\Delta t <3$). However, given the negligible probability that $|\Delta W|>A$ for the parameters used in Sects. 4.1.1 and 4.2.1, we did not observe any numerical issues, even though we did not use truncated increments. In the case of all the other numerical experiments presented in Sect. 4, the applied algorithms either turned out to be explicit, or the corresponding nonlinear systems of equations had solutions for all values of $\Delta W$. Nonlinear equations were solved using Newton’s method and the previous time step values of the position $q_k$ and momentum $p_k$ were used as initial guesses.

## Summary

In this paper we have presented a general framework for constructing a new class of stochastic symplectic integrators for stochastic Hamiltonian systems. We generalized the approach of Galerkin variational integrators introduced in [33, 40, 48] to the stochastic case, following the ideas underlying the stochastic variational integrators introduced in [8]. The solution of the stochastic Hamiltonian system was approximated by a polynomial of degree *s*, and the action functional was approximated by a quadrature formula based on *r* quadrature points. We showed that the resulting integrators are symplectic, preserve integrals of motion related to Lie group symmetries, and include stochastic symplectic Runge–Kutta methods introduced in [35, 36, 44] as a special case when $r=s$. We pointed out several new low-stage stochastic symplectic methods of mean-square order 1.0 for systems driven by a one-dimensional noise, both for the case of a general Hamiltonian function $h=h(q,p)$ and a Hamiltonian function $h=h(q)$ independent of *p*, and demonstrated their superior long-time numerical stability and energy behavior via numerical experiments. We also stated the conditions under which these integrators retain their first order of convergence when applied to systems driven by a multidimensional noise.

Our work can be extended in several ways. In Sect. 3.6 we indicated how higher-order stochastic variational integrators can be constructed and showed that a type of stochastic symplectic partitioned Runge–Kutta methods of mean-square order 3/2 considered in [44] can be recast in that formalism. It would be interesting to derive new stochastic integrators of order 3/2 by choosing appropriate values for the parameters in (3.54) or (3.56). It would also be interesting to apply the Galerkin approach to construct stochastic variational integrators for constrained (see [7]) and dissipative (see [9]) stochastic Hamiltonian systems, and systems defined on Lie groups (see [32]). Another important problem would be stochastic variational error analysis. That is, rather than considering how closely the numerical solution follows the exact trajectory of the system, one could investigate how closely the discrete Hamiltonian matches the exact generating function. In the deterministic setting these two notions of the order of convergence are equivalent (see [40]). It would be instructive to know if a similar result holds in the stochastic case. A further vital task would be to develop higher-order weakly convergent stochastic variational integrators. As mentioned in Sects. 3.1 and 3.6, higher-order methods require inclusion of higher-order multiple Stratonovich integrals, which are cumbersome to simulate in practice. In many cases, though, one is only interested in calculating the probability distribution of the solution rather than precisely approximating each sample path. In such cases weakly convergent methods are much easier to use (see [28, 42]). Finally, one may extend the idea of variational integration to stochastic multisymplectic partial differential equations such as the stochastic Korteweg–de Vries, Camassa–Holm or Hunter–Saxton equations. Theoretical groundwork for such numerical schemes has been recently presented in [24].

## Footnotes

1 In this work we only consider Hamiltonian functions *H* and *h* that are independent of time. In the time-dependent case one needs to add a further assumption that the growth of *A* and *B* is linearly bounded, i.e. $\Vert A(z,t)\Vert ^2+\Vert B(z,t)\Vert ^2 \le K (1 + \Vert z\Vert ^2)$ for a constant $K>0$ (see [5, 27–29]).

2 A generating function for the symplectic transformation $(q_a,p_a)\longrightarrow (q_b,p_b)$ is a function of one of the variables $(q_a,p_a)$ and one of the variables $(q_b,p_b)$. Therefore, there are four basic types of generating functions: $S=S_1(q_a, q_b)$, $S=S_2(q_a, p_b)$, $S=S_3(p_a, q_b)$, and $S=S_4(p_a, p_b)$. In this work we use the type-II generating function $S=S_2(q_a, p_b)$.

3 In analogy to ordinary calculus, the total stochastic differential is understood as $S_2(q_a, {\bar{p}}(t_b),t_b)-S_2(q_a, {\bar{p}}(t_a),t_a) = \int _{t_a}^{t_b} {\bar{d}}S_2(q_a, {\bar{p}}(t),t)$, whereas the partial stochastic differential means $S_2(q_a, p_b,t_b)-S_2(q_a, p_b,t_a) = \int _{t_a}^{t_b} dS_2(q_a,p_b,t)$.
